# Blood-based analysis of type-2 diabetes mellitus susceptibility genes identifies specific transcript variants with deregulated expression and association with disease risk

**DOI:** 10.1038/s41598-018-37856-1

**Published:** 2019-02-06

**Authors:** Maria-Ioanna Christodoulou, Margaritis Avgeris, Ioanna Kokkinopoulou, Eirini Maratou, Panayota Mitrou, Christos K. Kontos, Efthimios Pappas, Eleni Boutati, Andreas Scorilas, Emmanuel G. Fragoulis

**Affiliations:** 1 Department of Biochemistry and Molecular Biology, Faculty of Biology, National and Kapodistrian University of Athens, Athens, Greece; 2 Hellenic National Center for Research, Prevention and Treatment of Diabetes Mellitus and its Complications (HNDC), Athens, Greece; 3 Institute of Infection, Immunity and Inflammation, University of Glasgow, Glasgow, UK; 4 Second Department of Internal Medicine, School of Medicine, Attikon Hospital, National and Kapodistrian University of Athens, Athens, Greece

## Abstract

Despite significant progress by genome-wide association studies, the ability of genetic variants to conduce to the prediction or prognosis of type-2 diabetes (T2D) is weak. Expression analysis of the corresponding genes may suggest possible links between single-nucleotide polymorphisms and T2D phenotype and/or risk. Herein, we investigated the expression patterns of 24 T2D-susceptibility genes, and their individual transcript variants (tv), in peripheral blood of T2D patients and controls (CTs), applying RNA-seq and real-time qPCR methodologies, and explore possible associations with disease features. Our data revealed the deregulation of certain transcripts in T2D patients. Among them, the down-regulation of *CAPN10* tv3 was confirmed as an independent predictor for T2D. In patients, increased expression of *CDK5* tv2, *CDKN2A* tv3 or *THADA* tv5 correlated positively with serum insulin levels, of *CDK5* tv1 positively with % HbA1c levels, while in controls, elevated levels of *TSPAN8* were associated positively with the presence of T2D family history. Herein, a T2D-specific expression profile of specific transcripts of disease-susceptibility genes is for the first time described in human peripheral blood. Large-scale studies are needed to evaluate the potential of these molecules to serve as disease biomarkers.

## Introduction

Type-2 diabetes mellitus (T2D), a chronic metabolic disorder with increased cardiovascular morbidity and mortality, accounts currently for one of the global epidemics with ever growing prevalence^1^. Despite recent advances in T2D diagnosis and management, challenges in its prevention and treatment still remain^2^.

T2D epidemic is mainly ascribed to the continuous increase in obesity globally, favored nowadays by the adoption of a sedentary lifestyle^2^, while the risk for T2D development depends also on genetic components. During the last decade, over 60 genome-wide association studies (GWAS) revealed more than 250 single nucleotide polymorphisms (SNPs) related to T2D or glycemic traits^3^. However, each of them individually increases disease risk with rather modest effect sizes (25–40% increase in the homozygous state for the genes conveying the greatest risk)^4^, which are further weakened when introduced in multivariate analysis models^5^.

The implication of the genome in the development of human disorders can be elucidated through the study of the transcriptome, given that the last reflects functionality^6–9^. Recent advances in transcriptome analysis provide key-data for (i) the link between genotype and phenotype, (ii) molecular networks underlying pathophysiological processes, and (iii) molecular fingerprints, paving the way for the identification of possible therapeutic targets and/or disease biomarkers^7,10^. Next-generation RNA-sequencing (RNA-Seq) has pivotally fashioned the mode of transcriptome profiling, giving the chance for gene-transcription levels and splicing isoforms to be detected and quantitated, in a high-throughput manner^7,11,12^.

The gene-expression signature of T2D, including the expression patterns of T2D-susceptibility genes, has been hardly investigated. Previous studies were confined to pancreatic islets or beta-cell lines from animal models or deceased human donors^13^, mainly due to difficulties in obtaining biopsy specimens from the T2D-target tissue(s) of living donors. However, recent evidence support that the gene-expression profile of peripheral blood cells reflects significantly (>80%) the gene-expression profile of other tissues, including disease-affected tissues, and that changes in the former mirror changes in the micro- and macro-environment of the latter^14^. Thus, peripheral blood is considered as a reliable alternative for the investigation of transcriptome dynamics of organ-specific and systemic diseases, as it is easily accessible, and provides data for pathophysiological processes taking place in various sites throughout the human body^15^.

Herein, we investigated the expression patterns of highly-related T2D-susceptibility genes in peripheral blood samples of patients and controls and explored possible associations with disease parameters and risk factors.

## Materials and Methods

### Study design

First, we developed a panel of highly-associated T2D-susceptibility genes. For the quantification of their expression, appropriate reverse transcription (RT) - real-time PCR (qPCR) protocols were developed and applied on RNA extracted from whole peripheral blood samples of T2D patients and controls (CT). RNA-Seq and specific qPCR protocols were utilized to identify specific transcript variants of these genes that are differentially expressed between the two groups. To examine specific distribution patterns in individuals at high risk of developing the disease, a distinct group of controls bearing T2D-risk factors was included in the total group of controls. The two subgroups were analyzed both together and separately. Finally, possible associations between the gene or transcript-variant expression levels and various disease parameters were explored.

### Development of the T2D-susceptibility gene panel

The 24 highly-associated T2D-susceptibility gene panel was developed upon in-depth search in the NHGRI-EBI Catalog of published GWAS and SNPedia online databases^3,16^ (Table 1). *CAPN10* is not included in GWAS-significant genes, however, in SNPedia it presents as carrying variants related to T2D in different populations, and thus it was included in the panel. *CDK5* was also included, since it is highly regulated by the T2D-susceptibility gene *CDKAL1*^17^.

**Table 1:** T2D-susceptibility genes selected to be investigated in the current study, upon search in NHGRI-EBI Catalog of published GWAS and SNPedia online databases.

Gene symbol	Gene name	Chromosomal region	SNP	No of CS/GWAS report significance	*p*-value min	*p*-value max
*ADAMTS9*	ADAM metallopeptidase with thrombospondin type 1 motif 9	3p14.1	rs4607103	1	1.00e-08	—
*CAPN10*	Calpain 10	2q37.3	rs3792267	4	3.00e-03	3.00e-02
				2	1.00e-02	3.00e-02
			rs5030952	1	5.00e-02	—
*CDC123/CAMK1D*	Cell division cycle 123/ calcium/calmodulin dependent protein kinase ID	10p13	rs10906115	1	1.00e-08	
			rs12779790	1	1.00e-10	
			rs11257655	3	1.00e-12	7.00e-09
*CDKAL1*	CDK5 regulatory subunit associated protein 1 like 1	6p22.3	rs7766070	4	2.00e-11	9.00e-09
			rs7754840	4	2.00e-13	7.00e-10
			rs7756992	2	1.00e-16	8.00e-09
			rs10946398	2	1.00e-08	7.00e-07
			rs4712523	1	7.00e-20	—
			rs6931514	1	1.00e-11	—
			rs4712524	1	3.00e-10	—
			rs10440833	1	2.00e-22	—
			rs9295474	1	9.00e-06	—
			rs35612982	1	6.00e-36	—
			rs9465871	1	3.00e-07	—
*CDKN2A/CDKN2B*	Cyclin dependent kinase inhibitor 2A/cyclin dependent kinase inhibitor 2B	9p21.3	rs2383208	1	2.00e-29	—
			rs10811661	6	1.00e-27	5.00e-06
			rs564398	1	1.00e-06	—
			rs1333051	1	6.00e-10	—
			rs7020996	1	2.00e-07	—
			rs10965250	1	1.00e-10	—
			rs2383208	2	5.00e-33	3.00e-06
*CDK5*	Cyclin-dependent kinase 5		Regulated by *CDKAL1*			
*FTO*	FTO, alpha-ketoglutarate dependent dioxygenase	16q12.2	rs9939609	2	1.00e-20	2.00e-07
			rs8050136	5	2.00e-17	7.00e-06
			rs11642841	1	3.00e-08	—
			rs9936385	1	1.00e-12	—
			rs1421085	1	4.00e-15	—
*HHEX*	Hematopoietically expressed homeobox	10q23.33	rs5015480	5	1.00e-15	9.00e-06
			rs1111875	6	3.00e-19	3.00e-06
			rs78627331	1	2.00e-14	—
			rs34773007	1	2.00e-14	—
			rs7087591	1	6.00e-20	—
*HNF1B*	HNF1 homeobox B	17q12	rs4430796	4	2.00e-11	4.00e-06
			rs10908278	1	4.00e-15	—
*HNF4A*	Hepatocyte nuclear factor 4 alpha	20q13.12	rs4812829	2	3.00e-10	5.00e-08
			rs6017317	1	1.00e-11	—
*IGF2BP2*	Insulin-like growth factor 2 mRNA-binding protein 2	3q27.2	rs4402960	7	1.00e-17	1.00e-06
			rs1374910	1	1.00e-07	—
			rs1470579	8	2.00e-24	5.00e-06
			rs138306797	1	3.00e-06	—
			rs6769511	1	1.00e-09	—
			rs11927381	1	3.00e-14	—
*JAZF1*	JAZF zinc finger 1	7p15.1	rs864745	1	5.00e-14	—
			rs849134	2	6.00e-13	3.00e-09
			rs849135	1	2.00e-09	—
*KCNQ1*	Potassium voltage-gated channel subfamily Q member 1	11p15.4	rs2237892	5	2.00e-14	4.00e-06
			rs163182	1	2.00e-17	—
			rs2237895	1	1.00e-09	—
			rs2237897	2	1.00e-16	9.00e-15
			rs231362	1	3.00e-13	—
			rs2283228	2	5.00e-13	1.00e-10
			rs231356	1	4.00e-08	—
			rs8181588	1	5.00e-09	—
			rs163184	1	2.00e-14	—
			rs2237896	1	3.00e-70	—
			rs117601636	1	1.00e-07	—
*KCNJ11*	Potassium voltage-gated channel subfamily J member 11	11p15.1	rs5215	3	3.00e-11	4.00e-07
			rs5219	4	7.00e-11	5.00e-07
*MTNR1B*	Melatonin receptor 1B	11q14.3	rs1387153	1	8.00e-15	—
			rs10830963	1	2.00e-07	—
*NOTCH2*	Neurogenic locus notch homolog protein 2	1p12	rs10923931	1	4.00e-08	—
*PPARG*	Peroxisome proliferator activated receptor gamma	3p25.2	rs1801282	4	6.00e-10	2.00e-06
			rs17036101	1	2.00e-07	—
			rs13081389	1	2.00e-07	—
*SLC30A8*	Solute carrier family 30 member 8	8q24.11	rs13266634	10	2.00e-14	7.00e-06
			rs3802177	3	2.00e-18	4.00e-08
*TCF7L2*	Transcription factor 7 like 2	10q25.2	rs7903146	24	4.00e-94	5.00e-08
			rs7901695	2	1.00e-48	1.00e-06
			rs34872471	3	6.00e-53	8.00e-08
			rs4506565	1	5.00e-12	—
*THADA*	THADA, armadillo repeat containing	2p21	rs7578597	1	1.00e-09	—
*TSPAN8*	Tetraspanin 8	12q21.1	rs7961581	1	1.00e-09	—
			rs4760790	1	4.00e-06	—
			rs1495377	1	7.00e-06	—
*WFS1*	Wolframin ER transmembrane glycoprotein	4p16.1	rs1801214	1	3.00e-08	—
			rs4458523	1	2.00e-09	—

Annotations for the most highly T2D-associated SNPs in each gene, number of large-scale clinical (CS) or genome-wide association studies (GWAS) describing significant association of each SNP with the disease, as well as minimum and maximum *p*-values reported among studies, are stated (in the case of SNPs described in only one study, a sole *p*-value is stated).

### Patients and samples

Peripheral blood samples were collected from 48 consecutive T2D patients and 40 control (CT) individuals (with normal glucose metabolism), upon informed written consent. The study was approved by the Ethics Committee of the Attikon Hospital (Athens, Greece). Both groups were characterized according to the current criteria for T2D diagnosis^18^. The medical records of the participants were evaluated for various clinical, laboratory, and therapeutic variables. The group of controls consisted of two distinct subgroups: controls without risk factors for the development of T2D (CT_RF−_; n = 17) and controls bearing risk factors for the disease (CT_RF+_; n = 23), as these were previously described by Nathan^2^ (Table 2).

**Table 2:** Characteristics of control individuals (CT) and patients (T2D) included in the study.

Features		CT (n = 40)	T2D (n = 48)
*General*	Age (years); *median (range)*	50 (19–69)	60 (35–77)
	Sex (male/female); *number (%)*	21/19 (53/47)	27/21 (56/44)
	Disease duration (years); *median (range)*	NA	5 (0–26)
	Family history (yes/no); *number (%)*	16/24 (40/60)	34/14 (71/29)
	Risk factors^*^ (presence/absence); *number (%)*	23/17 (57/43)	NA
*Anthropometric*	BMI (body mass index)^†^; *median (range)*	27.0 (21.3–36.3)	29.5 (21.5–46.5)
	<25: normal weight; *number (%)*	18 (45)	7 (15)
	25–30: overweight; *number (%)*	14 (35)	19 (41)
	>30: obese; *number (%)*	8 (20)	22 (46)
	W/H (waist-to-hip ratio); *median (range)*	0.90 (0.71–1.09)	0.93 (0.83–1.18)
	Central obesity^‡^ (yes/no); *number (%)*	15/25 (38/62)	43/5 (90/10)
*Clinical*	Hypertension^§^ (yes/no); *number (%)*	5/35 (13/87)	29/19 (60/40)
	Hyperlipidemia^‖^ (yes/no); *number (%)*	8/32 (20/80)	37/11 (77/23)
	Metabolic syndrome^¶^ (yes/no); number (%)	6/34 (15/85)	37/11 (77/23)
*Laboratory*	HbA1c levels (% or mmol/ml); *median (range)*	5.6 (4.9–6.1)	6.6 (5.2–12.1)
	<7% or 53; *number (%)*	40 (100)	33 (69)
	≥7% or 53; *number (%)*	0 (0)	15 (31)
	Glucose levels (mg/dl); *median (range)*	85 (68–120)	
	<130; *number (%)*	40 (100)	29 (60)
	≥130; *number (%)*	0 (100)	19 (40)
	Insulin levels (μU/ml); *median (range)*	9.4 (5.2–19.1)	13.7 (3.8–56.0)
	Cholesterol levels (mg/dl); *median (range)*		
	Total cholesterol	199 (109–281)	184 (119–256)
	High-density cholesterol (HDL)	49 (6–79)	41 (17–125)
	Low-density cholesterol (LDL)	122 (19–192)	113 (53–191)
	Triglycerides levels (mg/dl); *median (range)*	114 (65–176)	147 (79–363)
*T2D therapy*	Naïve (prior to treatment); *number (%)*	NA	8 (16.7)
	Tablets (metformin, vildagliptin, glimeripide, gliclazide); *number (%)*	NA	22 (45.8)
	Two tablets (metformin + glimepiride, metformin + vildagliptin); or one tablet (metformin) + injectable GLP-1 (liraglutide) or injectable DPP-4 inhibitor (sitagliptin, saxagliptin); *number (%)*	NA	8 (16.7)
	Three tablets (metformin + vildagliptin + pioglitazone or metformin + vildagliptin + glimepiride); or two tablets (metformin + glimeripide) + injectable DPP-4 inhibitor (sitagliptin); *number (%)*	NA	4 (8.3)
	Injectable insulin (±tablets: metformin + sitagliptin); *number (%)*	NA	3 (6.3)
	Multiple injections of insulin; number *(%)*	NA	3 (6.3)

^*^Risk factors associated with higher risk of T2D, included: (i) BMI >25, (ii) prior history of gestational diabetes, (iii) hypertension, (iv) dyslipidemia, (v) cardiovascular disease, or vi) first-degree family member with T2D^2^. ^†^BMI was calculated as weight (kg) divided by the square of height (m^2^). ^‡^Central obesity was regarded if waist circumference was ≥102 cm (40 in) in men or ≥88 cm (35 in) in women. ^§^Hypertension was regarded if blood pressure was ≥130/85 mm Hg (or receiving drug therapy for hypertension); ^‖^Hyperlipidemia (defined by the Adult Treatment Panel III of the National Cholesterol Education Program^53^. ^¶^Metabolic syndrome was diagnosed according to the NCEP-ATP III report^54^ requiring at least 3 of the following 5 conditions: (i) fasting glucose ≥100 mg/dL (or receiving drug therapy for hyperglycemia), (ii) blood pressure ≥130/85 mm Hg (or receiving drug therapy for hypertension), (iii) triglycerides ≥150 mg/dL (or receiving drug therapy for hypertriglyceridemia), (iii) HDL-C <40 mg/dL in men or <50 mg/dL in women (or receiving drug therapy for reduced HDL-C), (iv) waist circumference ≥102 cm (40 in) in men or ≥88 cm (35 in) in women. NA: not applicable.

### RNA extraction, RT-qPCR and RNA-seq

All the methods used for gene-expression analysis, were applied on RNA extracted from whole peripheral blood using direct-blood lysis. For materials and protocols applied for RNA extraction, reverse transcription, and qPCR, as well as cDNA library construction and RNA-seq, see Supplemental Fig. 1. Specific primers designed for the amplification of the genes-of-interest, or certain transcript variants of them are reported in Supplemental Table 1. Relative quantification (RQ) of gene expression was performed by the 2^−ΔΔCt^ method, using the hypoxanthine phosphoribosyltransferase 1 (*HPRT1*) gene, as endogenous reference gene for normalization purposes, and the immortalized 1.2B4 pancreatic beta-cell line (ECACC, Salisbury, UK), as our assay calibrator, for the calculation of the fold-changes. Representative amplification, melting and standard curves for certain genes/transcript variants are indicatively presented in Supplemental Fig. 2.

### Bioinformatics analysis

For analysis of RNA-seq raw data see Supplemental Fig. 1. Differential expression between the two groups was considered significant if fold-change of their average RPKMs (CT:T2D ratio) was <0.5 or >2. Area-proportional Euler diagrams were generated using the BioVenn tool (http://www.biovenn.nl/). Analysis of tissue-specific expression patterns of genes and transcript variants and expression quantitative trait loci (eQTLs) was performed utilizing the portal of the Genotype-Tissue Expression (GTEx) project^19^ and the Blood eQTL browser^20^.

### Statistical analysis

Differential expression patterns between CT and T2D, or among CT_RF−_, CT_RF+_, and T2D individuals, were explored using the non-parametric Mann–Whitney *U* or Jonckheere-Terpstra tests, respectively. Benjamini-Hochberg procedures for adjusting the false discovery rate (FDR = 0.25) in multiple comparisons were also applied. Possible associations with binary, ordinal or continuous values of various clinicopathological and laboratory parameters were investigated by Mann-Whitney *U*, Jonckheere-Terpstra, or Spearman’s rank correlation coefficient tests, respectively. Binomial multivariate logistic regression analysis was performed (enter model) using the occurrence of T2D as the dependent variable and the expression levels of genes and transcript variants, age and sex as independent variables. Analyses were performed using the softwares: GraphPad Prism 5 (GraphPad Software Inc., San Diego, CA, USA) or IBM SPSS Statistics 21 (IBM Corp., Armonk, NY, USA). *P*-values < 0.05 were considered significant.

### Statement of Ethical Approval and Informed Consent

The study was approved by the Ethics Committee of the Attikon Hospital (Athens, Greece). All procedures followed were in accordance with the ethical standards of the responsible committee on human experimentation (institutional and national) and with the Helsinki Declaration of 1975, as revised in 2008 (5). Informed consent was obtained from all patients for being included in the study.

## Results

### Differential expression of certain T2D-susceptibility genes in patients *versus* controls

Firstly, specifically designed qPCR protocols applied on RNA extracted from whole peripheral blood samples, detected quantifiable expression levels in the cases of the following 20 genes: *CAMK1D*, *CAPN10*, *CDC123*, *CDK5*, *CDKAL1*, *CDKN2A*, *CDKN2B*, *FTO*, *HHEX*, *IGF2BP2*, *JAZF1*, *KCNJ11*, *KCNQ1*, *NOTCH2*, *PPARG*, *SLC30A8*, *TCF7L2*, *THADA*, *TSPAN8* and *WFS1*. On the contrary, mRNA expression was not detected in samples of either patients or controls, in the cases of *ADAMTS9*, *HNF1B*, *HNF4A* and *MTNR1B* genes.

**Figure 1:**
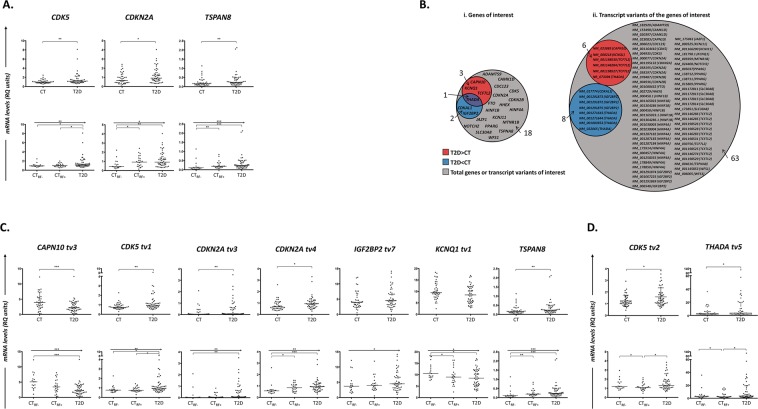
(**A**) Dot-plots depicting the differential distribution of mRNA levels (RQ units) of *CDK5*, *CDKN2A* and *TSPAN8* in controls (CT) and T2D patients (T2D) (*upper row of panels*), as well as among controls without T2D risk factors (CT_RF−_), controls with T2D risk factors (CT_RF+_) and T2D patients (*lower row of panels*), as attested by appropriate non-parametric tests. Mann-Whitney analysis revealed that T2D patients are characterized by higher mRNA levels of *CDK5*, *CDKN2A* and *TSPAN8*, compared to controls (*upper row*). Jonckheere-Terpstra test showed a stepwise increase in the total mRNA levels of the abovementioned genes among the CT_RF−_, CT_RF+_ and T2D groups (*lower row*). *P*-values are designated by asterisks (*p < 0.05, **p < 0.01, ***p < 0.001), whereas horizontal bars represent the median value of the group (**B**). Area-proportional Euler diagrams showing the differentially expressed genes and transcripts variants between the samples of controls (CT) and T2D patients (T2D), as analysed by RNA-seq. a. Diagrams representing the differentially expressed genes between T2D patients and controls, within the total 24 genes-of-interest (T2D-susceptibility genes; grey circle). Based on our data, T2D patients expressed exclusively higher levels of total mRNA of 3 genes (namely *CAPN10*, *KCNQ1*, *TCF7L2*) and lower levels of total mRNA of 2 genes (namely *CDKAL1* and *IGF2BP2*), compared to controls, while mRNA expression of one gene (*THADA*) was found to be either up- or- downregulated in T2D *versus* CT groups. Further analysis of the individual gene-transcript variants revealed that the levels of 6 out of the 77 transcript variants of interest [NM_023085 (*CAPN10*), NM_000218 (*KCNQ1*), NM_001198530, NM_001146284, NM_001198527 (all of *TCF7L2*), NR_073394 (*THADA*)] were increased, while 8 [NM_017774 (*CDKAL1*), NM_001291873, NM_001291872, NM_001291875 (all of *IGF2BP2*), NM_001271643, NM_001271644, NM_001083953, NM_022065 (all of *THADA*)] decreased in patients *versus* controls. Differential expression was considered as fold-change of the relative expression levels (mean of reads per kilobase million, RPKM) between the two groups (CT:T2D ratio) <0.5 or >2. (**C**) Dot-plots depicting the differential distribution of mRNA levels (RQ units) of *CAPN10 tv3*, *CDK5 tv1, CDKN2A tv3*, *CDKN2A tv4*, *IGF2BP2 tv7*, *KCNQ1 tv1* and *TSPAN8* in controls (CT) and T2D patients (T2D) (*upper row of panels*), as well as among controls without T2D risk factors (CT_RF−_), controls with T2D risk factors (CT_RF+_) and T2D patients (*lower row of panels*), as attested by appropriate non-parametric tests. Mann-Whitney analysis revealed that T2D patients are characterized by higher mRNA levels of *CDK5 tv1*, *CDKN2A tv3*, *CDKN2A tv4*, *IGF2BP2 tv7*, and *TSPAN8*, while lower levels of *CAPN10 tv3* and *KCNQ1 tv1*, compared to controls (*upper row*). Jonckheere-Terpstra test showed that the mRNA levels’ distribution of the abovementioned transcripts followed a linear trend among the CT_RF−_, CT_RF+_ and T2D groups: increase in the cases of *CDK5 tv1*, *CDKN2A tv3*, *CDKN2A tv4*, *IGF2BP2 tv7*, and *TSPAN8* and decrease in the cases of *CAPN10 tv3* and *KCNQ1 tv1* (*lower row*). *P*-values are designated by asterisks (*p < 0.05, **p < 0.01, ***p < 0.001), whereas horizontal bars represent the median value of the group; D. Dot-plots depicting the differential distribution of mRNA levels (RQ units) of *CDK5* tv2 and *THADA* tv5 in controls (CT) and T2D patients (T2D) (*upper row of panels*), as well as among controls without T2D risk factors (CT_RF−_), controls with T2D risk factors (CT_RF+_) and T2D patients (*lower row of panels*), as attested by appropriate non-parametric tests. Mann-Whitney analysis revealed that T2D patients are characterized by higher mRNA levels of *CDK5* tv2 and *THADA* tv5 (*upper row*). More specifically, there is a significant difference of these tv levels between T2D individuals and the group of controls with T2D risk factors (CT_RF+_), while not with the controls without such factors (*lower row*). *P*-values are designated by asterisks (*p < 0.05), whereas horizontal bars represent the median value of the group.

Relative quantification (RQ) values (median; range) in the groups of T2D patients (n = 48) and controls (n = 40) are summarized in Table 3. Mann-Whitney *U* test revealed that compared to controls, T2D patients expressed significantly higher levels of the genes *CDK5* [p = 0.0056, RQ values (median; range) for T2D = 1.151 (0.600–8.103) and for CT = 0.945 (0.512–2.473), fold-change T2D *vs*. CT = 1.22], *CDKN2A* [p = 0.0411, RQ values (median; range) for T2D = 0.910 (0.320–4.030) and for CT = 0.655 (0.150–2.420), fold-change T2D *vs*. CT = 1.39] and *TSPAN8* [p = 0.0055, RQ values (median; range) for T2D = 0.234 (0.0398–2.124) *vs*. CT = 0.159 (0.0247–1.132), fold-change T2D *vs*. CT = 1.47] (Table 3 and Fig. 1A; upper row). Further analysis within the group of CTs, revealed that CT_RF+_ individuals (n = 23) were characterized by elevated levels of the three abovementioned genes compared to CT_RF−_ (n = 17) ones (Table 3 and Fig. 1A; lower row). Jonckheere-Terpstra test further confirmed this increase, revealing a gradual up-regulation in the mRNA levels of these genes among the groups of CT_RF−_, CT_RF+_ and T2D subjects [as for the *CDK5* gene: p = 0.009, RQ values (median; range) for CT_RF−_ = 0.919 (0.625–2.473), for CT_RF+_ = 1.005 (0.512–1.998) and for T2D = 1.151 (0.600–8.103), fold-change for CT_RF+_ *vs*. CT_RF−_ = 1.09 and for T2D *vs*. CT_RF+_ = 1.15; as for the *CDKN2A* gene: p = 0.010, RQ values (median; range) for CT_RF−_ = 0.426 (0.200–2.220), for CT_RF+_ = 0.920 (0.150–2.420) and for T2D = 0.910 (0.320–4.030), fold-change for CT_RF+_ *vs*. CT_RF−_ = 2.16 and for T2D *vs*. CT_RF+_ = 0.99; as for the *TSPAN8* gene: p = 0.001, RQ values (median; range) for CT_RF−_ = 0.1071 (0.0247–1.132), for CT_RF+_ = 0.1894 (0.0741–0.832) and for T2D = 0.2340 (0.0398–2.124), fold-change for CT_RF+_ *vs*. CT_RF−_ = 1.77 and for T2D *vs*. CT_RF+_ = 1.24) (Table 3 and Fig. 1A; lower row). Statistics for all comparisons are reported in Table 3. After applying correction for multiple comparisons, the differential expression patterns that remained significant were those of: *CDK5* and *TSPAN8* between CT and T2D groups, and of *CDK5*, *CDKN2A* and *TSPAN8* among the CT_RF−_, CT_RF+_ and T2D groups.

**Table 3:** Relative quantification (RQ) expression levels of total variants of the 24 genes-of-interest and of their specific transcript variants.

Gene/Variant	RQ levels, median (range)				Statistical significance, *p*				
	CT_TOTAL_ (n = 40)	CT_RF−_ (n = 17)	CT_RF+_ (n = 23)	T2D (n = 48)	CT_TOTAL_ *vs*. T2D	CT_RF−_ *vs*. T2D	CT_RF+_ *vs*. T2D	CT_RF−_ *vs*. CT_RF+_	Linear trend following the CT_RF−_ → CT_RF+_ → T2D order
***Genes of interest (total variants)***									
*ADAMTS9*	NE	NE	NE	NE	NA	NA	NA	NA	NA
*CAMK1D*	**29.82** (9.467–61.03)	**30.24** (21.71–61.03)	**29.27** (9.467–49.79)	**33.66** (15.99–52.76)	0.9142	0.8178	0.7229	0.4455	0.907
*CAPN10*	**2.784** (0.968–5.987)	**2.785** (2.199–5.987)	**2.783** (0.968–5.721)	**2.699** (1.026–5.384)	0.4390	0.1306	0.9116	0.2881	0.193
*CDC123*	**4.859** (2.959–6.831)	**4.808** (3.196–6.019)	**4.937** (2.959–6.831)	**4.909** (3.069–11.59)	0.1160	0.2612	0.1711	0.9086	0.135
*CDK5*	**0.945** (0.512–2.473)	**0.919** (0.625–2.473)	**1.005** (0.512–1.998)	**1.151** (0.600–8.103)	**0.0056**	**0.0263**	**0.0264**	0.9943	**0.009**
*CDKAL1*	**4.311** (0.934–6.972)	**4.544** (2.545–6.440)	**4.214** (0.934–6.972)	**3.733** (0.652–8.709)	0.3256	0.1688	0.7639	0.3135	0.238
*CDKN2A*	**0.655** (0.150–2.420)	**0.426** (0.200–2.220)	**0.920** (0.150–2.420)	**0.910** (0.320–4.030)	**0.0411**	**0.0032**	NS	**0.0385**	**0.010**
*CDKN2B*	**0.596** (0.198–1.376)	**0.576** (0.379–1.376)	**0.615** (0.198–1.018)	**0.652** (0.155–1.819)	0.2766	0.4521	0.3519	0.9112	0.230
*FTO*	**1.583** (0.641–2.559)	**1.432** (0.734–2.352)	**1.780** (0.641–2.559)	**1.512** (0.320–2.638)	0.3015	0.9741	0.1235	0.2506	0.626
*HHEX*	**20.25** (5.30–37.23)	**20.00** (5.300–37.23)	**20.25** (5.30–33.41)	**23.50** (5.300–45.59)	0.1802	0.3541	0.2340	0.8076	0.145
*HNF1B*	NE	NE	NE	NE	NA	NA	NA	NA	NA
*HNF4A*	NE	NE	NE	NE	NA	NA	NA	NA	NA
*IGF2BP2*	**3.139** (0.443–12.57)	**3.670** (1.568–5.501)	**3.100** (0.443–12.57)	**3.671** (0.777–20.09)	0.2438	0.4521	0.3046	0.8953	0.143
*JAZF1*	**197.9** (12.46–688.8)	**214.9** (45.36–688.8)	**182.4** (12.46–498.3)	**173.9** (6.230–549.2)	0.7540	0.8944	0.6639	0.6765	0.956
*KCNJ11*	**0.536** (0.010–15.50)	**0.338** (0.010–15.50)	**0.568** (0.010–11.01)	**0.466** (0.010–43.85)	0.6220	0.1509	0.6551	0.9618	0.605
*KCNQ1*	**8.939** (2.083–15.48)	**9.363** (5.344–12.15)	**8.431** (2.083–15.48)	**7.760** (1.040–15.07)	0.2640	0.1509	0.6551	0.3687	0.191
*MTNR1B*	NE	NE	NE	NE	NA	NA	NA	NA	NA
*NOTCH2*	**5.228** (0.710–9.706)	**5.303** (0.710–9.706)	**5.153** (0.710–9.069)	**4.859** (0.710–11.94)	0.5411	0,5588	0,6850	0.9353	0.640
*PPARG*	**0.01533** (0.0012–0.0361)	**0.01540** (0.0012–0.0279)	**0.01520** (0.0012–0.0361)	**0.01475** (0.0012–0.0738)	0.8397	0.8562	0.8860	0.9622	0.856
*SLC30A8*	**5.094** (0.0–695.4)	**20.48** (1.001–695.4)	**3.075** (0.0–364.0)	**6.099** (0.0–3139)	0.3922	0.5728	0.1908	0.1574	0.796
*TCF7L2*	**3.803** (0.911–8.146)	**3.678** (1.942–8.146)	**3.827** (0.911–7.488)	**3.782** (1.673–10.13)	0.6388	0.6860	0.7138	0.7475	0.507
*THADA*	**1.867** (0.592–4.052)	**1.542** (0.592–3.985)	**1.951** (0.817–4.052)	**1.788** (0.362–3.962)	0.8096	0.6147	0.8160	0.7117	0.602
*TSPAN8*	**0.1590** (0.0247–1.132)	**0.1071** (0.0247–1.132)	**0.1894** (0.0741–0.832)	**0.2340** (0.0398–2.124)	**0.0055**	**0.0007**	0.1889	**0.0057**	**0.001**
*WFS1*	**0.2015** (0.0362–0.4887)	**0.2193** (0.0362–0.4887)	**0.1861** (0.0842–0.3805)	**0.2235** (0.0388–0.4155)	0.2984	0.8687	0.1603	0.3743	0.465
***Transcript variants of interest***									
*CAPN10* tv3	**3.924** (0.313–12.30)	**5.098** (1.016–12.30)	**3.405** (0.313–8.056)	**2.208** (0.313–12.43)	**0.0004**	<**0.0001**	**0.0521**	**0.0655**	<**0.0005**
*CDK5* tv1	**0.745** (0.285–2.266)	**0.754** (0.509–2.266)	**0.735** (0.285–1.441)	**0.943** (0.494–8.233)	**0.0034**	**0.0206**	**0.0177**	0.8196	**0.006**
*CDK5* tv2	**1.117** (0.453–2.938)	**1.190** (0.788–2.938)	**1.071** (0.453–2.139)	**1.308** (0.631–3.843)	**0.0367**	0.3732	**0.0196**	0.2098	0.167
*CDKN2A* tv1	NE	NE	NE	NE	NA	NA	NA	NA	NA
*CDKN2A* tv3	**0.0300** (0.0062–2.083)	**0.0145** (0.0062–2.083)	**0.0390** (0.0094–0.820)	**0.0722** (0.0062–3.481)	**0.0035**	**0.0022**	**0.0770**	**0.0579**	**0.002**
*CDKN2A* tv4	**0.669** (0.321–2.600)	**0.570** (0.321–2.600)	**0.850** (0.360–1.489)	**0.928** (0.292–3.300)	**0.0125**	**0.0002**	0.4922	**0.0151**	**0.001**
*CDKN2A* tv5	NE	NE	NE	NE	NA	NA	NA	NA	NA
*IGF2BP2* tv4	NE	NE	NE	NE	NA	NA	NA	NA	NA
*IGF2BP2* tv4, 5, 6 & 7	**0.0106** (0.0106–0.4114)	**0.0106** (0.0106–0.3017)	**0.0106** (0.0106–0.4114)	**0.0106** (0.0106–0.9255)	0.8008	0.8537	0.5898	0.6346	0.970
*IGF2BP2* tv7	**3.903** (0.5160–12.14)	**3.670** (1.4040–12.14)	**3.989** (0.5160–11.91)	**4.485** (1.032–14.14)	0.2167	0.1665	0.2076	0.5629	**0.050**
*KCNQ1* tv1	**9.408** (2.445–18.34)	**10.520** (7.504–18.34)	**8.951** (2.445–16.34)	**8.456** (2.880–16.93)	**0.0761**	**0.0143**	0.5530	**0.0448**	**0.013**
*TCF7L2* tv4 & 9	**1.478** (0.842–3.843)	**1.773** (0.842–3.843)	**1.476** (1.087–3.190)	**1.392** (0.396–4.554)	0.4641	0.6832	0.5051	0.8466	0.567
*TCF7L2* tv12	**3.036** (0.247–7.023)	**3.315** (1.434–6.988)	**2.808** (0.247–7.023)	**3.490** (0.494–7.614)	0.7903	0.3856	0.2659	0.1558	0.945
*THADA* tv1 & 3	**2.513** (1.256–5.316)	**2.736** (1.559–4.599)	**2.126** (1.256–5.316)	**2.326** (1.008–7.321)	0.5999	0.1509	0.6596	0.1302	0.382
*THADA* tv4	**2.319** (0.916–6.221)	**2.241** (1.519–6.221)	**2.397** (0.916–4.080)	**2.464** (0.912–8.510)	0.4336	0.9741	0.2606	0.4618	0.549
*THADA* tv5	**2.582** (0.596–35.83)	**2.933** (1.128–35.83)	**2.304** (0.596–16.01)	**3.452** (0.718–120.7)	**0.0479**	**0.0237**	**0.0341**	0.3544	0.129

Data are expressed as median values (range). The between-group up- or down-regulation and the CT_RF−_ → CT_RF+_ → T2D-ordered linear trend were evaluated by Mann-Whitney *U* and Jonckheere-Terpstra non-parametric tests, respectively.

### RNA-Seq analysis

Following the quantification of the expression levels of the abovementioned 20 genes, we studied also the expression patterns of their individual transcript variants in order to: (i) detect the specific transcript variant(s) responsible for the aforesaid differences and (ii) reveal any possible “hidden” differences in the levels of specific variants of the rest 17 genes, in patients *versus* controls. For that reason, data from RNA-Seq performed on peripheral blood samples of representative T2D patients (n = 4) and controls (n = 2) were analyzed appropriately.

Focusing on the 24 genes-of-interest, T2D patients were found to express: (i) higher levels of the genes *CAPN10*, *KCNQ1* and *TCF7L2* and of the transcripts NM_023085 (*CAPN10*), NM_000218 (*KCNQ1*), NM_001198530, NM_001146284, NM_001198527 (all of *TCF7L2*), NR_073394 (*THADA*) (Fig. 1B and Supplemental Table 2) and (ii) and lower levels of the genes *CDKAL1* and *IGF2BP2* and of the transcripts NM_017774 (*CDKAL1*), NM_001291873, NM_001291872, NM_001291875 (all of *IGF2BP2*), NM_001271643, NM_001271644, NM_001083953, NM_022065 (all of *THADA*) (Fig. 1B and Supplemental Table 2), compared to controls.

### Differential expression of certain transcript variants in T2D patients *versus* CT controls

After collecting together data from both the qPCR and RNA-Seq assays, we further evaluated in the total cohort of the study (48 T2D patients and 40 controls): (i) the levels of individual transcript variants of the genes found to be differentially expressed by qPCR (*CDK5*, *CDKN2A*, *TSPAN8*; Table 3 and Fig. 1A) and (ii) the levels of certain transcript variants found to be differentially expressed in RNA-Seq experiments (Fig. 1B and Supplemental Table 2). These transcript variants (tv) were the: NM_023085 (*CAPN10* tv3), NM_004935.3 and NM_001164410.2 (*CDK5* tv1 and 2, respectively), NM_000077.4; *p16INK4A*, NM_058197.4, NM_058195.3; *p14ARF*, and NM_001195132.1 (*CDKN2A* tv1, 3, 4 & 5, respectively), NM_001291872, NM_001291873 and NM_001291875 (*IGF2BP2* tv4, 5 and 7, respectively), NM_000218 (*KCNQ1* tv1), *TCF7L2* tv4, 9 and 12 (NM_001146284, NM_001198527 and NM_001198530, respectively), NM_022065, NM_001083953, NM_001271643 and NM_001271644 (*THADA* tv1, 3, 4 and 5, respectively). The *CDKAL1* and *TSPAN8* genes, which have only one tv each (NM_017774 and NM_004616.2, respectively) were studied in the series of qPCR experiments described above. The NR_073394 non-coding tv of the *THADA* gene, was not selected to be further studied.

The levels (median; range) of the transcript variants in patient and control groups are reported in Table 3. Αs attested by Mann-Whitney *U* test, compared to controls, T2D patients expressed lower levels of *CAPN10* tv3 [p = 0.0004, RQ levels (median; range) for T2D = 2.208 (0.313–12.43) and for CT = 3.924 (0.313–12.30), fold-change T2D *vs*. CT = 0.56] and of *KCNQ1* tv1 [p = 0.0761, RQ levels (median; range) for T2D = 8.456 (2.880–16.93) and for CT = 9.408 (2.445–18.34), fold-change T2D *vs*. CT = 0.89] (Table 3 and Fig. 1C; upper row). Jonckheere-Terpstra test strongly supported these findings by revealing a significant gradual decrease among the CT_RF−_, CT_RF+_ and T2D groups in the levels of *CAPN10* tv3 [p < 0.0005, RQ levels (median; range) for CT_RF−_ = 5.098 (1.016–12.30), for CT_RF+_ = 3.405 (0.313–8.056), for T2D = 2.208 (0.313–12.43), fold-change for CT_RF+_ *vs*. CT_RF−_ = 0.67 and for T2D *vs*. CT_RF+_ = 0.65] and *KCNQ1* tv1 [p = 0.013, RQ levels (median; range) for CT_RF−_ = 10.520 (7.504–18.34), for CT_RF+_ = 8.951 (2.445–16.34) and for T2D = 8.456 (2.880–16.93), fold-change for CT_RF+_ *vs*. CT_RF−_ = 0.85 and for T2D *vs*. CT_RF+_ = 0.95] (Table 3 and Fig. 1C; lower row). On the other hand, compared to controls, patients exhibited higher levels of *CDK5* tv1 [p = 0.0034, RQ levels (median; range) for T2D = 0.943 (0.494–8.233) and for CT = 0.745 (0.285–2.266), fold-change T2D *vs*. CT = 1.27], of *CDKN2A* tv3 [p = 0.0035, RQ levels (median; range) for T2D = 0.0722 (0.0062–3.481) and for CT = 0.0300 (0.0062–2.083), fold-change T2D *vs*. CT = 2.41), of *CDKN2A* tv4 (p = 0.0125, RQ levels (median; range) for T2D = 0.928 (0.292–3.300) and for CT = 0.669 (0.321–2.600), fold-change T2D *vs*. CT = 1.39) and of *IGF2BP2* tv7 (p = 0.22, RQ levels (median; range) for T2D = 4.485 (1.032–14.14) and for CT = 3.903 (0.516–12.14), fold-change T2D *vs*. CT = 1.15) (Table 3 and Fig. 1C; upper row). Also, a significant gradual increase in the levels of these transcripts was observed among the groups of CT_RF−_, CT_RF+_ and T2D patients [for *CDK5* tv1: p = 0.006, RQ levels (median; range) for CT_RF−_ = 0.754 (0.509–2.266), for CT_RF+_ = 0.735 (0.285–1.441) and for T2D = 0.943 (0.494–8.233), fold-change CT_RF+_ *vs*. CT_RF−_ = 0.97 and for T2D *vs*. CT_RF+_ = 1.28; for *CDKN2A* tv3: p = 0.002, RQ levels (median; range) for CT_RF−_ = 0.0145 (0.0062–2.083), for CT_RF+_ = 0.0390 (0.0094–0.820) and for T2D = 0.0722 (0.0062–3.481), fold-change for CT_RF+_ *vs*. CT_RF−_ = 2.67 and for T2D *vs*. CT_RF+_ = 1.85; for *CDKN2A* tv4: p = 0.001, RQ levels (median; range) for CT_RF−_ = 0.570 (0.321–2.600), for CT_RF+_ = 0.850 (0.360–1.489) and for T2D = 0.928 (0.292–3.300), fold-change for CT_RF+_ *vs*. CT_RF−_ = 1.49 and for T2D *vs*. CT_RF+_ = 1.09; for *IGF2BP2* tv7: p = 0.050, RQ levels (median; range) for CT_RF−_ = 3.670 (1.4040–12.14), for CT_RF+_ = 3.989 (0.5160–11.91) and for T2D = 4.485 (1.032–14.14), fold-change for CT_RF+_ *vs*. CT_RF−_ = 1.09 and for T2D *vs*. CT_RF+_ = 1.12) (Table 3 and Fig. 1C; lower row).

A different distribution pattern was detected in the case of *CDK5* tv2 and *THADA* tv5: T2D patients expressed elevated levels compared to controls [for *CDK5* tv2: p = 0.0367, RQ levels (median; range) for T2D = 1.308 (0.631–3.843) and for CT = 1.117 (0.453–2.938), fold-change T2D *vs*. CT = 1.17; for *THADA* tv5: p = 0.0479, RQ levels (median; range) for T2D = 3.452 (0.718–120.7) and for CT = 2.582 (0.596–35.83), fold-change = 1.34) (Table 3 and Fig. 1D; upper row), though, the lowest levels were detected in CT_RF+_ individuals and intermediate values in CT_RF−_ subjects [for *CDK5* tv2: RQ levels (median; range) for CT_RF−_ = 1.190 (0.788–2.938), for CT_RF+_ = 1.071 (0.453–2.139) and for T2D = 1.308 (0.631–3.843), fold-change for CT_RF+_ *vs*. CT_RF−_ = 0.90 and for T2D *vs*. CT_RF+_ = 1.22; for *THADA* tv5: RQ levels (median; range) for CT_RF−_ = 2.933 (1.128–35.83), for CT_RF+_ = 2.304 (0.596–16.01) and for T2D = 3.452 (0.718–120.7), fold-change CT_RF+_ *vs*. CT_RF−_ = 0.79 and for T2D *vs*. CT_RF+_ = 1.50] (Table 3 and Fig. 1D; lower row).

Moreover, correction for multiple comparisons revealed statistically significant differences in the levels of *CAPN10* tv3, *CDK5* tv1, *CDK5* tv2, *CDKN2A* tv3, *CDKN2A* tv4, and *THADA* tv5 between controls and T2D patients, and of *CAPN10* tv3, *CDK5* tv1, *CDKN2A* tv3, *CDKN2A* tv4, *IGF2BP2* tv7, *KCNQ1* tv1 among CT_RF−_, CT_RF+_ and T2D subjects.

**Figure 2:**
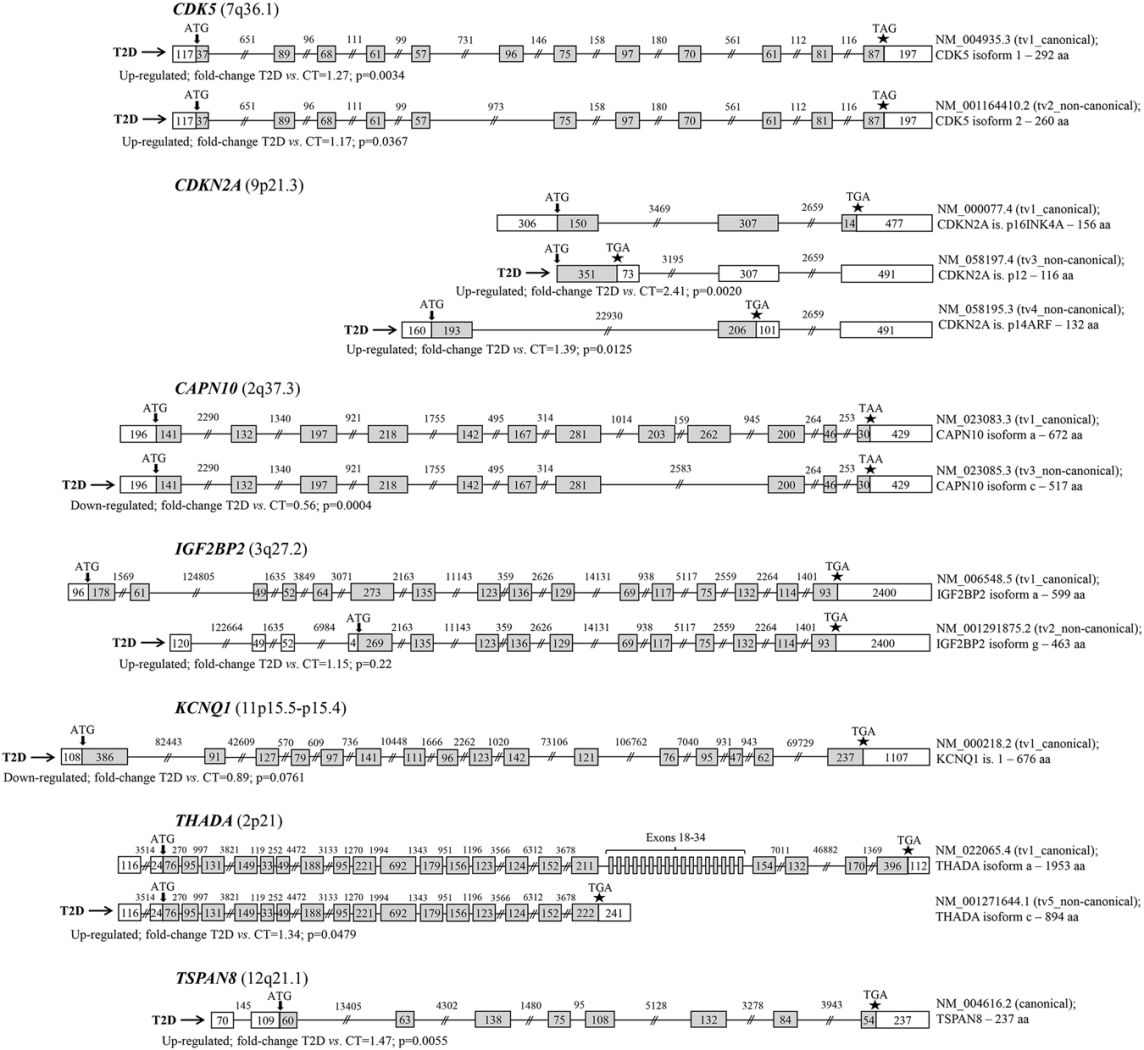
Detailed gene-structure of the transcript (splice) variants of *CDK5*, *CDKN2A*, *CAPN10*, *IGF2BP2*, *KCNQ1*, *THADA* and *TSPAN8* that were found to be differentially expressed in T2D. Exons are presented as boxes and introns as lines. Grey and white boxes represent coding and non-coding exons, respectively. The numbers within the boxes and above the lines indicate exon’s or intron’s length in nucleotides (nt). Arrows (**↓**) and asterisks (*) indicate the positions of the ATG starting codon and the stop codon (TGA or TAA or TAG), respectively. In each gene, the canonical (classic) and the differentially expressed transcript variants in T2D patients, indicated by arrow (**→**) are depicted. For each transcript variant, the GenBank^®^ accession number, as well as the protein isoform and length in amino acids (aa) are shown.

Based on the above findings, the panel of the T2D-specific transcript variants finally included the: *CAPN10* tv3, *CDK5* tv1, *CDK5* tv2, *CDKN2A* tv3, *CDKN2A* tv4, *IGF2BP2* tv7, *KCNQ1* tv1, *THADA* tv5 and *TSPAN8*. Among them, binomial multivariate analysis corrected for age and sex revealed that *CAPN10* tv3 can predict T2D among participants of the current study (p = 0.022, OR = 0.726). A schematic representation of the T2D-specific transcript variants, also in comparison with the canonical transcript for each gene, is shown in Fig. 2.

### Associations of mRNA levels with clinicopathological data

The levels of the T2D-specific transcripts were found to associate with various clinicopathological parameters (Supplemental Table 3). Notably, the associations revealed were different in patient *versus* control groups.

In detail, *CDK5* tv1 levels correlated positively with serum insulin (μU/ml) and glycated haemoglobin (HbA1c; % or mmol/mol) levels and negatively with hyperlipidemia in T2D patients, while positively with serum triglycerides levels (mg/dl) (p < 0.05) in CT_RF+_ subjects. As for the *CDK5* tv2 levels, these also correlated strongly with serum insulin levels, and moreover with the presence of central obesity in the T2D group (p < 0.05).

The levels of *CDKN2A* tv3 were as well significantly associated with serum insulin levels in T2D patients (p < 0.01), while these of *CDKN2A* tv4 correlated positively with BMI and waist-to-hip ratio and negatively with serum HDL levels (mg/dl) in the CT_RF+_ subgroup (p < 0.05).

Serum insulin levels in T2D patients associated also with *THADA* tv5 (p < 0.01) and *TSPAN8* levels (p < 0.05), tended to correlate negatively with *KCNQ1* tv1 levels (p = 0.06), while, in CT_RF+_ subjects, associated with *IGF2BP2* tv7 levels (p < 0.05). Additionally, in T2D individuals, the levels of *THADA* tv5 correlated reversely with hyperlipidemia and those of *IGF2BP2* tv7 positively with BMI (p < 0.05). In CT_RF+_ individuals, mRNA levels of *TSPAN8* associated with T2D family history (p < 0.01), of *IGF2BP2* tv7 with serum glucose levels, while in the total group of controls, *KCNQ1* tv1 levels reversely with BMI, central obesity, glucose and LDL (mg/dl) levels (p < 0.05).

Furthermore, correction for multiple comparisons confirmed certain of the above correlations: a) In T2D subjects, serum insulin levels were associated with the levels of *CDK5* tv1, *CDK5* tv2, *CDKN2A* tv3, *KCNQ1* tv1, *THADA* tv5 and *TSPAN8*, hyperlipidemia was associated with the levels of *CDK5* tv1 and *THADA* tv5, and BMI with the levels of *IGF2BP2* tv7; b) In control individuals, serum insulin levels were correlated with the levels of *IGF2BP2* tv7, serum HDL levels, BMI, and waist-to-hip ratio with the levels of *CDKN2A* tv4, and family history of T2D with the levels of *TSPAN8*.

Various associations were also detected between clinicopathological data and the levels of transcripts which did not exhibit any differential distribution among the groups of patients and controls (data not shown).

### Analysis of the tissue-specific expression pattern and eQTLs of the differentially expressed genes using public available datasets

**Figure 3:**
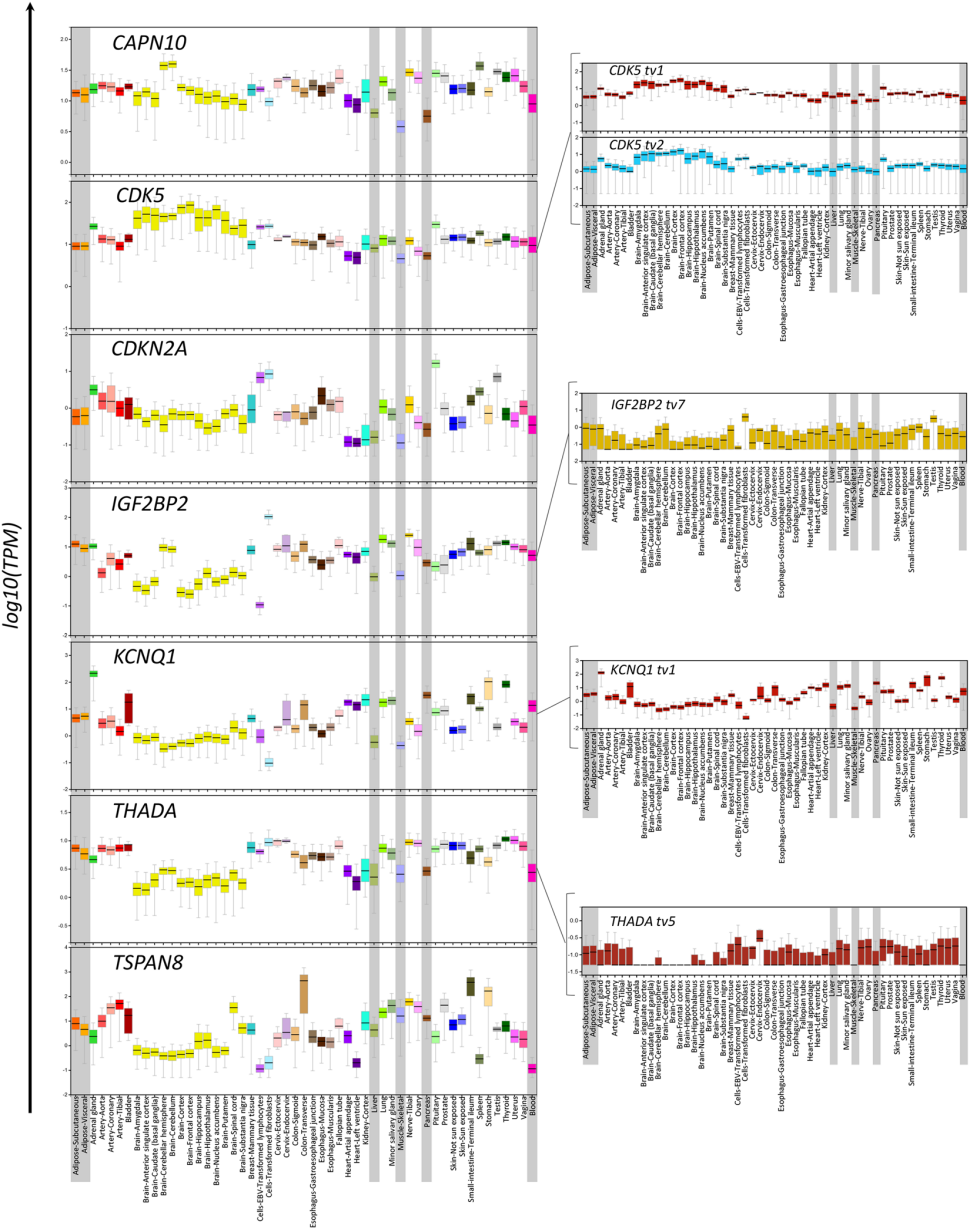
Bar diagrams obtained from the GTEx portal (5/3/2018) depicting the expression pattern of the differentially expressed genes and transcript variants in various human tissues and cells. mRNA levels are expressed as transcripts per million (TPM). Boxes represent the median (range) of the normalized values. Boxes corresponding to whole blood or T2D-affected tissues are grey-colored highlighted.

Based on public available data of the GTEx portal^19^, the *CAPN10*, *CDK5*, *CDKN2A*, *IGF2BP2*, *KCNQ1*, *THADA* and *TSPAN8* genes, as well as their transcript variants *CDK5* tv1, *CDK5* tv2, *IGF2BP2* tv7, *KCNQ1* tv1 and *THADA* tv5, are expressed in a series of human tissues including blood and T2D-target tissues (adipose tissue, liver, skeletal muscle, pancreas) (Fig. 3). No data were available for *CAPN10* tv3, *CDKN2A* tv3 and tv4.

Blood eQTL browser and GTEx portal were used for the analysis of eQTLs in the differentially expressed genes. For each one, a significant number of eQTLs appeared; however, we focused on eQTLs linked to T2D-related SNPs in blood or T2D-target tissues (Supplemental Table 4). Significant eQTLs related to T2D-SNPs were found on *CAPN10*, *CDKN2A*, *IGF2BP2* and *KCNQ1* in blood cells, and on *CAPN10* and *TSPAN8* in skeletal muscle. Affected genes are reported in Supplemental Table 4.

Also, *CAPN10*, the most significantly differentiated gene herein, is suggested to be an eGene (that is a gene having at least one *cis*-eQTL acting upon it), affected by several eQTLs on *GPR35*, *RNPEPL1*, and/or itself. Nevertheless, only the rs5030952-eQTL has a known (GWAS) association with T2D development (Supplemental Table 5).

## Discussion

Despite numerous GWAS and other clinical studies revealing a large pool of SNPs associated with T2D, none of them has been yet proven promising for its diagnosis and/or prognosis^5^. Moreover, their causal relationship with T2D pathogenesis is not well-defined; epigenetic mechanisms can influence the expression of T2D-susceptibility genes, while DNA sequence itself alters the way the epigenome exerts its regulatory effect^21–25^. Furthermore, eQTLs associated with T2D-genetic variants may be also involved^26^. Herein, the expression profile of a panel of T2D-susceptibility genes, with special focus on their individual transcripts, was investigated in peripheral blood of T2D patients and controls, utilizing a combination of high-throughput and highly-sensitive molecular methodologies (RNA-Seq and qPCR).

Our data revealed a T2D-specific pattern of expression of nine transcript variants of the genes: *CAPN10*, *CDK5*, *CDKN2A, IGF2BP2, KCNQ1, THADA* and *TSPAN8*. Compared to controls, patients exhibited down-regulated levels of the tv3 of *CAPN10* and tv1 of *KCNQ1*, while up-regulated levels of *CDK5* tv1 and tv2, *CDKN2A* tv3 and tv4, *IGF2BP2* tv7, *THADA* tv5 and *TSPAN8*. Publicly available datasets suggest that many human tissues including peripheral blood and T2D-target tissues express the abovementioned genes, allowing to postulate that the T2D-specific expression pattern found herein, may reflect pathogenetic mechanisms in disease-affected organs and/or peripheral blood^14,15,19,20^.

Among the down-regulated molecules is the tv3 of *CAPN10*. The gene encodes a protein implicated in glucose transporter 4 (GLUT4) translocation, insulin secretion, and apoptotic processes in pancreatic cells^27^. Compared to the canonical variant 1, tv3 lacks two consecutive exons, resulting in the loss of an in-frame segment in the 3’ coding region, and the encoded isoform (c) is shorter than isoform a (Fig. 2). The gene bears the rs3792267 and rs5030952 T2D-related SNPs^3,16^; however, tv3 does not bear any of them. Therefore, the decreased expression levels observed in patients, might be due to epigenetic and/or other transcriptional regulations^24,25^. It is worth mentioning that, herein, *CAPN10* tv3 exhibited the highest association with the disease among all the deregulated molecules, as attested by both univariate and multivariate analysis. Yet, it is the only transcript which showed no association with any of the clinicopathological parameters tested, possibly indicating the highly complicated molecular networks underlying T2D.

The levels of the canonical transcript (tv1) of *KCNQ1* (Fig. 2) were also decreased in the T2D group, while they were lower in the CT_RF+_ compared to the CT_RF−_ group. *ΚCNQ1* encodes the pore-forming subunit of a voltage-gated K^+^ channel (KvLQT1) that is essential for the repolarization phase of the action potential in cardiac muscle^28^. It is also expressed by pancreatic islets^29^, plays a key-role in the regulation of insulin secretion^30^ and its genetic variants have been associated with impaired insulin secretion in humans^31,32^. In these terms, the decreased expression of *KCNQ1* tv1 in the CT_RF+_ and T2D groups observed in our study, and the reverse correlation with serum insulin levels, may indeed reflect the negative regulation on insulin secretory function exerted by KCNQ1 in patients and pre-disposed individuals.

Among the T2D-up-regulated molecules, there are both the two transcripts of *CDK5* (Fig. 2). CDK5 is a serine/threonine protein kinase, involved in the degeneration of beta-cells and obstruction of insulin secretion through the generation of p35/CDK5 complexes^33^; its inhibition has been shown to protect these cells from glucotoxicity^34^. The overactivity of CDK5 and its activator p35 have been as well correlated with neuronal dysfunction in patients with Alzheimer’s disease (AD), and this could be one of the possible common mechanisms shared by these two degenerative disorders^34^. CDK5 is highly regulated by the T2D-susceptibility gene *CDKAL1*^17^, through the rs7756992 SNP of the latter, which increases the risk for T2D^35^. Our data showed decreased levels of *CDK5* tv1 and 2 in T2D patients compared to controls; though they exhibited different distribution patterns and correlated with different clinicopathological parameters in the CT_RF−_ *versus* CT_RF+_ groups, but both with increased serum insulin levels. This might be probably attributed to different transcriptional regulations and distinct pathogenetic mechanisms leading, however, to the same “pre-disease” phenotype. Moreover, it is reported that as tv1, the tv2 of *CDK5*, in which an in-frame coding exon is skipped^36^ (Fig. 2), is also a negative regulator of Wnt/β-catenin signalling, a pathway involved in T2D development^37^.

SNPs in the *CDKN2A/B* locus were recently implicated in the negative regulation of beta-cell mass, proliferation and insulin secretory function, as well as in metabolic processes in adipose tissue, liver and muscles^22^. Also, in human islets, this locus is affected by epigenetic factors^38^, however, no effect on gene expression is known^22^. *CDKN2A/B* variants affect also the risk for cardiovascular disease^39^ and cancer^40^, and this could be a link for the common pathogenetic mechanisms shared with T2D^41^. Additionally, in blood, T2D-associated SNPs on *CDKN2A* are eQTLs which affect the expression of *PSEN1*^19^ involved in AD and cancer^42,43^, connecting possibly these three morbidities. In this study, T2D patients expressed elevated levels of *CDKN2A*, and specifically of *CDKN2A* tv3 and 4: the first one highly associated with serum insulin levels in patients, and the second one with certain T2D-risk factors in controls. This may suggest their differential implication in disease development and/or progress. However, tv3 contains an alternative open reading frame (Fig. 2) and it is specifically expressed in the pancreas^44^. *CDKN2A* tv4 has a distinct first, but shares a common second exon with the canonical tv1, translated in different reading frames (Fig. 2): the encoded protein (p14ARF) lacks sequence similarity to the classic isoform (p16INK4a), and it is known to be nucleoplasmic but also recruited to mitochondria^45^. These characteristics may suggest tv-specific functions, possibly implicated in disease’s pathogenesis.

IGF2BP2 binds the 5′ UTR of the insulin-like growth factor 2 mRNA and regulates its translation^16^. Moreover, T2D-related SNPs on *IGF2BP2* are eQTLs affecting *SENP2*, a gene crucially involved in adipogenesis and T2D development^46^. Herein, the levels of the tv7 of *IGF2BP2* (which lacks exons 1 and 2 compared to the canonical tv1) exhibited a significant stepwise up-regulation from CT_RF−_ to CT_RF+_ and to T2D individuals. Their correlation with BMI in patients, and serum glucose and insulin levels in CT_RF+_ cases, indicates its functional involvement in T2D pathogenesis.

Patients exhibited also elevated levels of *TSPAN8* (only one known tv) and of *THADA* tv5 (with alternative 3′ coding region and 3′ UTR, encoding a shorter isoform (c) with a distinct C-terminus) (Fig. 2). The first gene is regarded as a prognostic factor and potential therapeutic target for certain human carcinomas^47–50^, while chromosomal aberrations of the second are observed in benign thyroid adenomas^51^. They both bear SNPs associated with T2D, though there is no knowledge regarding their involvement in its development^3,16^. Herein, the correlation of their levels with T2D, certain parameters and/or risk factors provides the first evidence for their possible implication in T2D pathogenesis.

The levels of *TCF7L2*, the most highly-related T2D-susceptibility gene, as well as of other T2D-susceptibility genes, were comparable between patients and controls; however, they correlated with certain disease characteristics or risk factors, supporting their implication in T2D development.

However, certain limitations of this study need to be considered: (a) the fact that not all the known T2D-susceptibilty genes are examined, (b) the relatively small number of participants and RNA-seq samples tested; the latter was overcome by the subsequent qPCR validation of the proposed deregulated transcript variants, discriminating between the true- and false-positive results, though, RNA-seq false-negative results could not been ruled out, (c) no paired-analysis of both the transcript levels and the presence of T2D-related SNPs was conducted; search throughout the Blood eQTL browser and the GTEx portal served only as a guide for their association and does not adequately explore the genetic determinants of the gene-expression variation, (d) the significant difference in the median age of patients and controls tested (Table 2); age associates with epigenetic changes^52^, thus it cannot be excluded as possible factor influencing the gene-expression variations observed in our cohort. However, after binomial multivariate analysis corrected for age and sex, *CAPN10* tv3 still remain capable to predict T2D.

Nevertheless, by analyzing the expression patterns of a panel of the most highly-associated T2D-susceptibility genes, the current study offers suggestive data on the deregulated levels of certain transcript variants. Future research is required to elucidate their involvement in principal molecular and biochemical networks underlying T2D pathogenesis. Also, large-scale perspective clinical studies are needed to evaluate their potential to serve as possible biomarkers for its diagnosis, prognosis and/or monitoring.

## Supplementary information

Supplementary Tables and Figures

## Data Availability

The RNA-seq and qPCR raw data used to support the findings of this study are available from the corresponding author upon request.
