# Supplementary material for: Blood-based analysis of type-2 diabetes mellitus susceptibility genes identifies specific transcript variants with deregulated expression and association with disease risk
Source: Sci Rep. 2019 Feb 6;9:1512. doi: 10.1038/s41598-018-37856-1 (PMC6365563; doi:10.1038/s41598-018-37856-1)
Supplement: Supplementary file 1 — Supplementary Tables and Figures [file 41598_2018_37856_MOESM1_ESM.pdf]

Blood-based analysis of type-2 diabetes mellitus susceptibility genes identifies specific transcript variants with deregulated expression and association with disease risk

Maria-Ioanna Christodoulou, Margaritis Avgeris, Ioanna K. Kokkinopoulou, Eirini Maratou, Panayota Mitrou, Christos K. Kontos, Efthimios Pappas, Eleni Boutati, Andreas Scorilas, Emmanuel G. Fragoulis

Supplemental Table 1. Pairs of primers used in qPCR for the amplification of genes and transcript variants of interest. Primers were designed based on published sequences (NCBI Reference Sequence) using the Primer Premier software (PREMIER Biosoft International, Palo Alto CA, U.S.A.). For each gene, a pair of specific primers was designed for the quantification of total transcript variants (mRNAs) levels. Non-coding transcript variants were excluded from amplification. Furthermore, a pair of primers was also designed for the quantification of each specific transcript variant of interest, as these were indicated by RNA-seq data analysis. Variants amplified, exons of hybridization, sequence, length (nucleotides; nt), % content in GC, melting temperature (Tm; ^{0}C) and final reaction concentration (nM) for each primer, as well as the length of the PCR product (base pairs; bp) in each case, are reported.

Gene / Coding transcript variants amplified (accession numbers)	Forward primer						Reverse primer						Product length (bp)
	Exon of hybridization	Sequence (5'→3')	Length (nt)	GC content (%)	Tm (^{0}C)	C (nM)	Exon of hybridization	Sequence (5'→3')	Length (nt)	GC content (%)	Tm (^{0}C)	C (nM)	
***ADAMTS9* total variants**<br>NM_182920.1 (tv1)<br>NM_001318781.1 (tv4)	31st	GCAACCCTGCGAGTATGTCTG	21	57.1	61.3	200	32nd	GGCAGTTGATGGTGGTTTGGT	21	52.4	61.4	200	152
***CAMK1D* total variants**<br>NM_020397.3 (tv1)<br>NM_153498.3 (tv2)	8th	CCTGATGGAGAAGGACCCGA	20	60.0	60.7	200	9th	GCTGACGGACTCGTGGATGT	20	60.0	62.2	200	109
***CAPN10* total variants**<br>NM_023083.3 (tv1)<br>NM_023085.3 (tv3)	4th	CTGAAGGGCGTAGCAGGAAG	20	60.0	60.5	200	5th	CGAGACAATGAAGGCATGGAA	21	47.6	58.6	200	171
***CAPN10* tv3**<br>NM_023085.3	7th-10th junction	GCACCTCTGGAAGGTCCCA	19	63.2	61.2	200	10th	CTCCTGGGCGTAGCGATGT	19	63.2	61.8	200	103
***CDC123* total variants**<br>NM_006023.2	5th	CGGGGGCAGTGTCTTTCCTA	20	60.0	61.6	200	6th	AGAAAGATGTCGCTGAGGGTTT	22	45.5	60.0	200	105
***CDK5* total variants**<br>NM_004935.3 (tv1)<br>NM_001164410.2 (tv2)	7th	TTCCCGTCCGCTGTTACTCA	20	55.0	61.2	150	8th	TCGATGGACGTGGAGTACAGC	21	57.1	61.9	150	94
***CDK5* tv1**<br>NM_004935.3	6th	GGGCTGGGATTCTGTCATAGC	21	57.1	60.5	150	8th	GATGGACGTGGAGTACAGCTTG	22	54.6	61.0	150	213
***CDK5* tv2**<br>NM_001164410.2	5th-7th junction	TCCTGAGATTGTAAAGAATGGGGAG	25	44.0	60.1	200	8th	TCGATGGACGTGGAGTACAGC	21	57.1	61.9	200	159
***CDKAL1* total variants**<br>NM_017774.3	9th	CTGCAAAACTAAACACGCCAGA	22	45.5	60.0	200	10th	CCCGTGTCTTCACTGGTCAAC	21	57.1	61.1	200	123
***CDKN2A* total variants**<br>NM_000077.4 (tv1)<br>NM_058197.4 (tv3)<br>NM_058195.3 (tv4)<br>NM_001195132.1 (tv5)	2nd	GCGATGTCGCACGGTACCT	19	63.2	62.7	200	3rd	CTGTAGGACCTTCGGTGACTGAT	23	52.2	61.4	200	166
***CDKN2A* tv1 & 5**<br>NM_000077.4 (tv1); *p16INK4A*<br>NM_001195132.1 (tv5)	1st	TGGAGCCTTCGGCTGACTG	19	63.2	61.9	200	2nd	GCCCATCATCATGACCTGGAT	21	52.4	60.0	200	140
***CDKN2A* tv3**<br>NM_058197.4	1st	CCCAACGCACCGAATAGTTACG	22	54.5	61.9	200	1st	TTCCCCTGCAAACTTCGTCC	20	55.0	60.5	200	108
***CDKN2A* tv4**<br>NM_058195.3; *p14ARF*	1st	TGAGGGTTTTCGTGGTTCACA	21	47.6	60.1	200	1st	CCCATCATCATGACCTGGTCTTCT	24	50.0	62.0	200	149

***CDKN2B* total variants**<br>NM_004936.3 (tv1)<br>NM_078487.2 (tv2)	1st	GTGTCGTTAAGTTTACGGCCAA	22	45.5	59.5	150	1st-2nd Junction	CCCATCATCATGACCTGGATC	21	52.4	57.9	150	233
***FTO* total variants**<br>NM_001080432.2	7th	ACTGGAAGCACTGTGGAAGAAGA	23	47.8	61.8	200	8th	TCAAGATTTCATTCCTTTGTTCCA	24	33.3	57.0	200	101
***HHEX* total variants**<br>NM_002729.4	1st	GCGGACGGTGAACGACTACA	20	60.0	62.5	200	2nd	GATGGTCTGGTCGTTGGAGAAT	22	50.0	60.0	200	139
***HNF1B* total variants**<br>NM_000458.3 (tv1)<br>NM_001165923.3 (tv2)<br>NM_001304286.1 (tv3)	4th	CTCCAACCAGACTCACAGCCT	21	57.1	61.7	200	5th	GGTGACTGATTGTTGAGGAGGAA	23	47.8	60.2	200	149
***HNF4A* total variants**<br>NM_178849.2 (tv1)<br>NM_000457.4 (tv2)<br>NM_178850.2 (tv3)<br>NM_001030003.2 (tv4)<br>NM_175914.4 (tv5)<br>NM_001030004.2 (tv6)<br>NM_001258355.1 (tv7)<br>NM_001287182.1 (tv8)<br>NM_001287183.1 (tv9)<br>NM_001287184.1 (tv10)	8th	TCCATACGCATCCTTGACGAG	21	52.4	59.9	200	9th	GGCGGTCGTTGATGTAGTCC	20	60	60.8	200	187
***HPRT1* total variants**<br>NM_000194.2	2nd	TGGAAAGGGTGTTTATTCCTCAT	23	39.1	57.7	300	3rd	ATGTAATCCAGCAGGTCAGCAA	22	45.5	60.0	300	151
***IGF2BP2* total variants**<br>NM_006548.5 (tv1)<br>NM_001007225.2 (tv2)<br>NM_001291869.2 (tv3)<br>NM_001291872.2 (tv4)<br>NM_001291873.2 (tv5)<br>NM_001291874.2 (tv6)<br>NM_001291875.2 (tv7)	15th	GAAGTCATCGTGCCTCGTGAC	21	57.1	61.3	200	16th	AGGCGACTCCCTGAGGGTAT	20	60.0	62.0	200	160
***IGF2BP2* tv4**<br>NM_001291872.2	1st	GACACTACCACGTTGATGGCT	21	52.4	60.3	200	5th	TGTGTTGACTAGGGAAAAGGCA	22	45.5	59.8	200	161
***IGF2BP2* tv7**<br>NM_001291875.2	4th	AATGTGGAACAAGAGCCATGGAG	23	47.8	61.1	100	6th	GCAGCGGGAAATCAATCTGTCT	22	50.0	61.3	100	198
***IGF2BP2* tv4, 5, 6 & 7** [a]<br>NM_001291872.2 (tv4)<br>NM_001291873.2 (tv5)<br>NM_001291874.2 (tv6)<br>NM_001291875.2 (tv7)	1st	TGGACACTACCACGTTGATGG	21	52.4	60.0	300	6th	GTTCTCAAACTGATGCCCGC	20	55.0	59.8	300	174
***JAZF1* total variants**<br>NM_175061.3	4th	GGACCACAGAGAGTGCCATCA	21	57.1	61.8	100	5th	TTCTGTGACCATTCTTAGCGTGA	23	43.5	60.0	100	156
***KCNJ11* total variants**<br>NM_001166290.1 (tv1)	1st	CAACAGCCCACTCTACGACCT	21	57.1	61.8	150	1st	CACTTTGACGGTGTTGCCAA	20	50.0	59.6	150	229

NM_000525.3 (tv2)													
***KCNQ1* total variants**<br>NM_000218.2 (tv1)<br>NM_181798.1 (tv2)	12nd	GGTCATTCGACGCATGCAGT	20	55.0	61.4	200	14th	TGAGGGCTTCCCAATGGACT	20	55.0	60.9	200	169
***KCNQ1* tv1**<br>NM_000218.2	1st	AATGCTTCGTTTACCACTTCGC	22	45.5	60.1	200	2nd	ATCCAGAAGAGAGTCCCCGT	20	55.0	59.7	200	115
***MTNR1B* total transcripts**<br>NM_005959.3	1st	TCCTGGTGATCCTCTCCGTG	20	60.0	60.7	200	2nd	GCCCAGCCGTCATAGAAGAT	20	55.0	59.6	200	145
***NOTCH2* total variants**<br>NM_024408.3 (tv1)<br>NM_001200001.1 (tv2)	34th	TTTGGCAACTAACGTAGAAACTCAAC	26	38.5	60.5	200	34th	TGCCAAGAGCATGAATACAGAGA	23	43.5	59.8	200	145
***PPARG* total variants**<br>NM_138712.3 (tv1)<br>NM_015869.4 (tv2)<br>NM_138711.3 (tv3)<br>NM_005037.5 (tv4)<br>NM_001330615.1 (tv5)	5th	TTGCAGTGGGGATGTCTCATAA	22	45.5	59.4	200	6th	TCAGCGGGAAGGACTTTATGTAT	23	43.5	59.3	200	174
***SLC30A8* total variants**<br>NM_173851.2 (tv1)<br>NM_001172814.1 (tv2)<br>NM_001172811.1 (tv3)<br>NM_001172813.1 (tv4)<br>NM_001172815.2 (tv5)	11th	AGGTGACTTGGCAAACACACAA	22	45.5	61.1	200	11th	TCCCTCCATAATGATTACCTGACA	24	41.7	58.8	200	124
***TCF7L2* total variants**<br>NM_001146274.1 (tv1)<br>NM_030756.4 (tv2)<br>NM_001146283.1 (tv3)<br>NM_001146284.1 (tv4)<br>NM_001146285.1 (tv5)<br>NM_001146286.1 (tv6)<br>NM_001198525.1 (tv7)<br>NM_001198526.1 (tv8)<br>NM_001198527.1 (tv9)<br>NM_001198528.1 (tv10)<br>NM_001198529.1 (tv11)<br>NM_001198530.1 (tv12)<br>NM_001198531.1 (tv13)	6th	AACCCACCTCCACACTTACCAG	22	54.5	61.9	200	7th	CTACGGTGCCAGGCGATAGT	20	60.0	61.7	200	109
***TCF7L2* tv4 & 9** [a]<br>NM_001146284.1 (tv4)<br>NM_001198527.1 (tv9)	6th	CCCTCACGCCTCTTATCACG	20	60.0	60.3	200	7th-8th<br>Junction	ACTGGTTGACCTTGCCATCCTA	22	50.0	61.4	200	196
***TCF7L2* tv12**<br>NM_001198530.1	3rd-6th<br>junction	CGCCCGAACCTCTAACAAAGT	21	52.4	60.6	200	7th	TGCCAGGCGATAGTGGGTAAT	21	52.4	61.3	200	203
***THADA* total variants**<br>NM_022065.4 (tv1)<br>NM_001083953.1 (tv3)<br>NM_001271643.1 (tv4)	12th	TGGGAGAACCTCGTGTCTGATG	22	54.6	61.7	200	13th	CTGGAGACTGGCTGTTAAGATTGT	24	45.8	60.6	200	181

NM_001271644.1 (tv5)													
***THADA* tv1 & 3** [a]<br>NM_022065.4 (tv1)<br>NM_001083953.1 (tv3)	35th	CTACACCACTTTTCCTCACCAACC	24	50.0	61.5	200	36th-37th junction	AGGCAAACTCTGTTGACTGGCA	22	50.0	62.7	200	180
***THADA* tv4**<br>NM_001271643.1	17th	GCTGCTGTGGTGGAAAGGAAC	21	57.1	62.0	200	18th	TGGATTTCTGAGACAGGAGGCA	22	50.0	61.1	200	206
***THADA* tv5**<br>NM_001271644.1	16th-17th junction	ACATTTTCAGGATTCGGGGAAAC	23	43.5	59.5	200	17th	AAGAGCAAACATCAATACAAACCCA	25	36.0	59.7	200	243
***TSPAN8* total variants**<br>NM_004616.2	2nd	TTCCGAAATGGCAGGTGTGA	20	50.0	59.9	150	4th	CAGCAATCAATATGTCCACAGCA	23	43.4	59.6	150	191
***WFS1* total variants**<br>NM_006005.3 (tv1)<br>NM_001145853.1 (tv2)	4th	GGCGACACGGATGAAGAACTC	21	57.1	61.3	100	5th	CCTCACCTCCCGTTCGTTCT	20	60.0	61.5	100	150

[a] In the case of transcript variants of *IGF2BP2*: highly-sensitive protocols were developed for the specific quantification of the expression levels of each of the transcript variants 4 or 7; nevertheless, appropriate and specific primers for the transcript variant 5 of *IGF2BP2* were not able to be designed, and thus the developed qPCR protocol amplified also the transcript variants 4, 6 and 7 of the same gene. Similarly, *TCF7L2* transcript variants 4 and 9 levels or *THADA* transcript variant 1 and 3 levels were quantified in one common protocol.

Supplemental Table 2. Data from RNAseq analysis in peripheral blood samples of two representative CT and four representative T2D individuals, regarding the transcript variants of the 24 genes-of- interest (T2D-susceptibility genes). Raw data of the reads and their normalized values (reads per kilobase million, RPKMs) in each sample, their means in CT and T2D groups as well as fold-change of RPKM in CT versus T2D groups (CT:T2D ratio) are reported. Differential expression was considered significant if fold-change was <0,5 (T2D upregulated versus CT; red-highlighted data) or >2 (T2D downregulated versus CT; blue-highlighted data).

	Rank	Chromosome	Start	Stop	Strand	Transcript	Transcript Length	Mean of Reads		Mean of RPKM		Fold-change of RPKM	Reads						RPKM					
								CT	T2D	CT	T2D		CT_S33	CT_S40	T2D_S11	T2D_S36	T2D_S53	T2D_S69	CT_S33	CT_S40	T2D_S11	T2D_S36	T2D_S53	T2D_S69
	ADAMTS9																							
1	37785	3	64515655	64687690	-	NM_182920	7312	NA	NA	**NA**	**NA**	**NA**	NA	NA	NA	NA	NA	NA	NA	NA	NA	NA	NA	NA
	CAMK1D																							
2	5550	10	12349543	12835546	+	NM_153498	8092	51.323	46.517	**6.298**	**5.345**	**1.178**	45.328	57.317	31.000	44.750	55.386	54.933	4.707	7.889	7.105	4.172	5.958	4.144
3	5549	10	12349543	12826465	+	NM_020397	2089	4.177	2.983	**1.987**	**1.075**	**1.849**	3.672	4.683	0.000	5.250	3.614	3.067	1.477	2.497	0.000	1.896	1.506	0.896
	CAPN10																							
4	32955	2	240586716	240599110	+	NM_023083	2644	5.000	3.231	**1.589**	**1.064**	**1.494**	10.000	0.001	0.000	0.000	12.922	0.000	3.178	0.000	0.000	0.000	4.255	0.000
5	32956	2	240586716	240599110	+	NM_023085	2179	6.000	2.019	**3.066**	**1.282**	**2.392**	0.000	11.999	5.000	0.000	0.078	3.000	0.000	6.133	4.256	0.000	0.031	0.840
	CDC123																							
6	5548	10	12195962	12250591	+	NM_006023	1548	26.500	20.000	**15.621**	**14.594**	**1.070**	39.000	14.000	27.000	19.000	20.000	14.000	21.170	10.072	32.350	9.259	11.247	5.521
	CDK5																							
7	51358	7	151053812	151057966	-	NM_001164410	1097	0.492	0.738	**0.499**	**0.469**	**1.065**	0.000	0.984	0.000	0.000	0.984	1.968	0.000	0.999	0.000	0.000	0.781	1.095
8	51359	7	151053812	151057966	-	NM_004935	1193	0.008	0.012	**0.008**	**0.007**	**1.065**	0.000	0.016	0.000	0.000	0.016	0.032	0.000	0.015	0.000	0.000	0.012	0.017
	CDKAL1																							
9	44170	6	20534457	21232404	+	NM_017774	3271	1.000	2.250	**0.299**	**0.776**	**0.385**	1.000	1.000	3.000	1.000	3.000	2.000	0.257	0.340	1.701	0.231	0.798	0.373
	CDKN2A																							
10	53680	9	21967752	21975134	-	NM_000077	1254	0.007	0.011	**0.005**	**0.007**	**0.715**	0.014	0.000	0.000	0.000	0.028	0.014	0.009	0.000	0.000	0.000	0.019	0.007
11	53681	9	21967752	21975134	-	NM_001195132	1451	0.000	0.000	**0.000**	**0.000**	**0.715**	0.000	0.000	0.000	0.000	0.000	0.000	0.000	0.000	0.000	0.000	0.000	0.000
12	53682	9	21967752	21994492	-	NM_058195	1151	0.468	0.702	**0.342**	**0.478**	**0.715**	0.936	0.000	0.000	0.000	1.872	0.936	0.683	0.000	0.000	0.000	1.416	0.497
13	53683	9	21967752	21974828	-	NM_058197	1222	0.025	0.037	**0.017**	**0.024**	**0.715**	0.050	0.000	0.000	0.000	0.100	0.050	0.034	0.000	0.000	0.000	0.071	0.025
	CDKN2B																							
14	53699	9	22002903	22009314	-	NM_078487	3984	0.177	0.221	**0.043**	**0.049**	**0.880**	0.177	0.177	0.177	0.177	0.000	0.531	0.037	0.049	0.082	0.034	0.000	0.081
15	53698	9	22002903	22009314	-	NM_004936	3861	0.823	1.029	**0.208**	**0.237**	**0.880**	0.823	0.823	0.823	0.823	0.000	2.469	0.179	0.237	0.395	0.161	0.000	0.390
	FTO																							
16	19420	16	53703963	54114468	+	NM_001080432	4292	7.000	7.000	**1.670**	**1.664**	**1.004**	5.000	9.000	5.000	4.000	13.000	6.000	0.703	2.637	0.979	0.703	2.637	2.335
	HHEX																							
17	6750	10	92689924	92695652	+	NM_002729	1759	8.000	10.500	**4.677**	**6.167**	**0.758**	5.000	11.000	11.000	12.000	9.000	10.000	2.389	6.965	11.599	5.146	4.454	3.471
	HINF1B																							
18	21596	17	37686431	37745079	-	NM_000458.1	2809	NA	NA	**NA**	**NA**	**NA**	NA	NA	NA	NA	NA	NA	NA	NA	NA	NA	NA	NA
19	21597	17	37686431	37745079	-	NM_001165923	2731	NA	NA	**NA**	**NA**	**NA**	NA	NA	NA	NA	NA	NA	NA	NA	NA	NA	NA	NA
20	21598	17	37686431	37745079	-	NM_001304286	2417	NA	NA	**NA**	**NA**	**NA**	NA	NA	NA	NA	NA	NA	NA	NA	NA	NA	NA	NA
21	23563	17	1925499	1984135	-	NM_000458	2809	NA	NA	**NA**	**NA**	**NA**	NA	NA	NA	NA	NA	NA	NA	NA	NA	NA	NA	NA
22	23564	17	1925499	1984135	-	NM_001165923.1	2731	NA	NA	**NA**	**NA**	**NA**	NA	NA	NA	NA	NA	NA	NA	NA	NA	NA	NA	NA
23	23565	17	1925499	1984135	-	NM_001304286.1	2417	NA	NA	**NA**	**NA**	**NA**	NA	NA	NA	NA	NA	NA	NA	NA	NA	NA	NA	NA
	HINF4A																							
24	33867	20	44355801	44432846	+	NM_001030003	4513	NA	NA	**NA**	**NA**	**NA**	NA	NA	NA	NA	NA	NA	NA	NA	NA	NA	NA	NA
25	33868	20	44355801	44424637	+	NM_001030004	1449	NA	NA	**NA**	**NA**	**NA**	NA	NA	NA	NA	NA	NA	NA	NA	NA	NA	NA	NA
26	33869	20	44355801	44432846	+	NM_001287182	4735	NA	NA	**NA**	**NA**	**NA**	NA	NA	NA	NA	NA	NA	NA	NA	NA	NA	NA	NA
27	33870	20	44355801	44432846	+	NM_001287183	4765	NA	NA	**NA**	**NA**	**NA**	NA	NA	NA	NA	NA	NA	NA	NA	NA	NA	NA	NA
28	33871	20	44355801	44424637	+	NM_001287184	1671	NA	NA	**NA**	**NA**	**NA**	NA	NA	NA	NA	NA	NA	NA	NA	NA	NA	NA	NA
29	33872	20	44355801	44432846	+	NM_175914	4543	NA	NA	**NA**	**NA**	**NA**	NA	NA	NA	NA	NA	NA	NA	NA	NA	NA	NA	NA
30	33874	20	44401256	44432846	+	NM_000457	4722	NA	NA	**NA**	**NA**	**NA**	NA	NA	NA	NA	NA	NA	NA	NA	NA	NA	NA	NA
31	33875	20	44401256	44432846	+	NM_001258355	4813	NA	NA	**NA**	**NA**	**NA**	NA	NA	NA	NA	NA	NA	NA	NA	NA	NA	NA	NA
32	33876	20	44401256	44432846	+	NM_178849	4692	NA	NA	**NA**	**NA**	**NA**	NA	NA	NA	NA	NA	NA	NA	NA	NA	NA	NA	NA
33	33877	20	44401256	44424637	+	NM_178850	1628	NA	NA	**NA**	**NA**	**NA**	NA	NA	NA	NA	NA	NA	NA	NA	NA	NA	NA	NA

	IGF2BP2																							
34	39140	3	185643739	185821132	-	NM_001291873	3473	0.701	2.773	**0.189**	**0.568**	**0.333**	0.905	0.496	0.056	7.243	0.026	3.766	0.219	0.159	0.030	1.573	0.007	0.662
35	39139	3	185643739	185821132	-	NM_001291872	3491	0.544	2.152	**0.146**	**0.439**	**0.333**	0.702	0.385	0.043	5.623	0.020	2.923	0.169	0.123	0.023	1.215	0.005	0.511
36	39142	3	185643739	185821132	-	NM_001291875	3409	1.743	6.898	**0.479**	**1.439**	**0.333**	2.251	1.235	0.138	18.020	0.065	9.368	0.555	0.404	0.075	3.988	0.017	1.678
37	39141	3	185643739	185821132	.	NM_001291874	3344	2.891	6.910	**0.727**	**1.300**	**0.559**	5.782	0.000	0.000	3.564	0.014	24.062	1.453	0.000	0.000	0.804	0.004	4.393
38	39137	3	185643739	185825040	-	NM_001007225	3542	6.253	3.697	**1.484**	**0.852**	**1.741**	12.492	0.015	0.000	3.676	9.678	1.436	2.964	0.005	0.000	0.783	2.379	0.248
39	39138	3	185643739	185825040	-	NM_001291869	3689	3.465	5.095	**0.983**	**1.412**	**0.696**	1.703	5.226	3.859	5.669	10.656	0.196	0.388	1.578	1.940	1.159	2.515	0.032
40	39143	3	185643739	185825040	-	NM_006548	3671	4.403	6.475	**1.255**	**1.803**	**0.696**	2.165	6.642	4.904	7.205	13.542	0.249	0.495	2.015	2.478	1.480	3.211	0.041
	JAZF1																							
41	49444	7	27830574	28180819	-	NM_175061	3199	42.000	47.250	**12.314**	**13.993**	**0.880**	54.000	30.000	40.000	37.000	33.000	79.000	14.185	10.444	23.191	8.725	8.980	15.076
	KCNJ11																							
42	8105	11	17385249	17388660	-	NM_000525	3411	NA	NA	**NA**	**NA**	**NA**	NA	NA	NA	NA	NA	NA	NA	NA	NA	NA	NA	NA
43	8106	11	17385249	17389332	-	NM_001166290	2756	NA	NA	**NA**	**NA**	**NA**	NA	NA	NA	NA	NA	NA	NA	NA	NA	NA	NA	NA
	KCNQ1																							
44	7707	11	2444991	2849111	+	NM_000218	3246	18.730	7.974	**5.395**	**2.576**	**2.094**	24.499	12.962	10.138	9.476	0.006	12.274	6.342	4.447	5.793	2.202	0.002	2.308
45	7708	11	2461454	2849111	+	NM_181798.1	3013	3.270	4.776	**1.186**	**1.365**	**0.869**	0.501	6.038	0.862	0.524	13.994	3.726	0.140	2.232	0.531	0.131	4.043	0.755
	MTNR1B																							
46	9795	11	92969623	92982783	+	NM_005959	1662	NA	NA	**NA**	**NA**	**NA**	NA	NA	NA	NA	NA	NA	NA	NA	NA	NA	NA	NA
	NOTCH2																							
47	2733	1	119911553	120069704	-	NM_024408	11466	177.000	159.687	**15.297**	**12.340**	**1.240**	158.999	195.000	77.000	122.000	241.000	198.749	11.653	18.941	12.455	8.026	18.298	10.582
	PPARG																							
48	36763	3	12287850	12434357	+	NM_005037	1818	0.438	0.000	**0.202**	**0.000**	**NA**	0.876	0.000	0.000	0.000	0.000	0.000	0.405	0.000	0.000	0.000	0.000	0.000
49	36764	3	12287850	12434357	+	NM_138712	1892	0.062	0.000	**0.028**	**0.000**	**NA**	0.124	0.000	0.000	0.000	0.000	0.000	0.055	0.000	0.000	0.000	0.000	0.000
50	36765	3	12288937	12434357	+	NM_138711	1919	NA	NA	**NA**	**NA**	**NA**	NA	NA	NA	NA	NA	NA	NA	NA	NA	NA	NA	NA
51	36766	3	12351502	12434357	+	NM_015869	1820	NA	NA	**NA**	**NA**	**NA**	NA	NA	NA	NA	NA	NA	NA	NA	NA	NA	NA	NA
	SLC30A8																							
52	52928	8	116950273	117176715	+	NM_001172811	5316	NA	NA	**NA**	**NA**	**NA**	NA	NA	NA	NA	NA	NA	NA	NA	NA	NA	NA	NA
53	52929	8	116950951	117176715	+	NM_001172813	5561	NA	NA	**NA**	**NA**	**NA**	NA	NA	NA	NA	NA	NA	NA	NA	NA	NA	NA	NA
54	52930	8	116950951	117176715	+	NM_001172815	5430	NA	NA	**NA**	**NA**	**NA**	NA	NA	NA	NA	NA	NA	NA	NA	NA	NA	NA	NA
55	52931	8	117135098	117176715	+	NM_001172814	5403	NA	NA	**NA**	**NA**	**NA**	NA	NA	NA	NA	NA	NA	NA	NA	NA	NA	NA	NA
56	52932	8	117135098	117176715	+	NM_173851	5373	NA	NA	**NA**	**NA**	**NA**	NA	NA	NA	NA	NA	NA	NA	NA	NA	NA	NA	NA
	TCF7L2																							
57	7189	10	112950250	113167678	+	NM_001198530	3777	3.678	1.405	**1.018**	**0.359**	**2.839**	1.839	5.517	1.021	0.001	2.758	1.839	0.409	1.627	0.501	0.000	0.636	0.297
58	7181	10	112950250	113167678	+	NM_001146284	3918	0.610	0.403	**0.163**	**0.078**	**2.085**	0.305	0.916	0.000	0.847	0.458	0.305	0.065	0.260	0.000	0.163	0.102	0.048
59	7183	10	112950250	113167678	+	NM_001146286	3879	0.997	0.726	**0.269**	**0.162**	**1.659**	0.498	1.495	0.277	1.383	0.747	0.498	0.108	0.429	0.132	0.269	0.168	0.078
60	7182	10	112950250	113167678	+	NM_001146285	3952	0.400	0.291	**0.106**	**0.064**	**1.659**	0.200	0.600	0.111	0.555	0.300	0.200	0.042	0.169	0.052	0.106	0.066	0.031
61	7185	10	112950250	113167678	+	NM_001198526	3952	0.400	0.291	**0.106**	**0.064**	**1.659**	0.200	0.600	0.111	0.555	0.300	0.200	0.042	0.169	0.052	0.106	0.066	0.031
62	7187	10	112950250	113167678	+	NM_001198528	4028	0.157	0.115	**0.041**	**0.025**	**1.659**	0.079	0.236	0.044	0.218	0.118	0.079	0.016	0.065	0.020	0.041	0.025	0.012
63	7186	10	112950250	113167678	+	NM_001198527	4016	0.182	0.120	**0.047**	**0.023**	**2.085**	0.091	0.273	0.000	0.253	0.136	0.091	0.019	0.076	0.000	0.047	0.030	0.014
64	7180	10	112950250	113167678	+	NM_001146283	4020	0.173	0.126	**0.045**	**0.027**	**1.659**	0.087	0.260	0.048	0.241	0.130	0.087	0.018	0.072	0.022	0.045	0.028	0.013
65	7190	10	112950250	113167678	+	NM_001198531	3948	0.420	0.306	**0.111**	**0.067**	**1.659**	0.210	0.630	0.117	0.583	0.315	0.210	0.045	0.178	0.055	0.111	0.069	0.032
66	7191	10	112950250	113167678	+	NM_030756	4003	0.213	0.155	**0.056**	**0.034**	**1.659**	0.107	0.320	0.059	0.296	0.160	0.107	0.022	0.089	0.027	0.056	0.035	0.016
67	7184	10	112950250	113167678	+	NM_001198525	4091	0.073	0.054	**0.019**	**0.011**	**1.659**	0.037	0.110	0.020	0.102	0.055	0.037	0.008	0.030	0.009	0.019	0.012	0.005
68	7179	10	112950250	113167678	+	NM_001146274	4021	0.171	0.125	**0.044**	**0.027**	**1.659**	0.086	0.257	0.047	0.238	0.128	0.086	0.018	0.071	0.022	0.045	0.028	0.013
69	7188	10	112950250	113167678	+	NM_001198529	3930	0.525	0.383	**0.140**	**0.084**	**1.659**	0.263	0.788	0.146	0.729	0.394	0.263	0.056	0.223	0.069	0.140	0.087	0.041
	THADA																							
70	30371	2	43552068	43595975	-	NM_001271643	3298	0.000	0.000	**0.000**	**0.000**	**0.000**	0.000	0.000	0.000	0.000	0.000	0.000	0.000	0.000	0.000	0.000	0.000	0.000
71	30372	2	43556093	43596047	-	NM_001271644	3066	0.000	0.000	**0.000**	**0.000**	**0.000**	0.000	0.000	0.000	0.000	0.000	0.000	0.000	0.000	0.000	0.000	0.000	0.000
72	30368	2	43230836	43595975	-	NM_001083953	6326	0.352	1.182	**0.054**	**0.151**	**0.354**	0.384	0.320	0.224	0.192	2.461	1.851	0.051	0.056	0.066	0.023	0.339	0.179
73	30369	2	43230836	43596047	-	NM_022065	6114	1.869	6.281	**0.295**	**0.833**	**0.354**	2.039	1.699	1.189	1.020	13.077	9.838	0.280	0.310	0.361	0.126	1.862	0.982
74	30370	2	43230836	43596047	-	NR_073394	5924	8.779	3.037	**1.429**	**0.651**	**2.194**	9.577	7.981	5.587	4.789	1.462	0.311	1.358	1.500	1.749	0.610	0.215	0.032
	TSPNA8																							

**75**	12306	12	71125097	71158000	-	NM_004616	1130	NA	NA	**NA**	**NA**	**NA**	NA	NA	NA	NA	NA	NA	NA	NA	NA	NA	NA	
	WFS1																							
**76**	39662	4	6269850	6303266	+	NM_001145853	3636	0.257	0.385	**0.059**	**0.083**	**0.715**	0.513	0.000	0.000	0.000	1.027	0.513	0.119	0.000	0.000	0.000	0.246	0.086
**77**	39663	4	6269850	6303266	+	NM_006005	3640	0.243	0.365	**0.056**	**0.079**	**0.715**	0.487	0.000	0.000	0.000	0.973	0.487	0.112	0.000	0.000	0.000	0.233	0.082

NA: not applicable; in the cases where Reads=0 in all the samples tested

Supplemental Table 3. Analysis of correlations between the mRNA levels of the transcript variants with differential distribution among the CT_{RF-}, CT_{RF+} and T2D groups, and certain clinical and laboratory features. Analysis was applied in the CT_{RF-} and CT_{RF+} subgroups separately, the total CT group and the T2D group, using appropriate non-parametric tests as required: differential expression levels in the cases of categorical variables [namely metabolic syndrome, central obesity, family history, hypertension, hyperlipidemia (presence; *p* or absence; *ab* of the manifestation), HbA1c levels (<7%=53 mmol/mol or >7%=53 mmol/mol) and glucose levels (<130 mg/dl or >130 mg/dl)] were evaluated by the Mann-Whitney test, in the cases of ordinal variables [namely BMI levels (<25, 25-30, >30)] by the Jonckheere-Terpstra test, while correlations of the mRNA expression levels with continuous variables [namely levels of BMI, W/H ratio, HbA1c, glucose, total cholesterol, HDL, LDL and triglycerides] were evaluated by the Spearman rank correlation test. The statistical significance (*p*), the mRNA expression levels [median (range)] in the groups of categorical and ordinal variables, and the regression (*r*) for continuous variables examined are displayed.

Variable	Type		*CAPN10* tv3				*CDK5* tv1				*CDK5* tv2			
			CT_{RF-}	CT_{RF+}	CT	T2D	CT_{RF-}	CT_{RF+}	CT	T2D	CT_{RF-}	CT_{RF+}	CT	T2D
**BMI**	*ordinal*	***p***	NA	0.975	0.283	0.940	NA	0.759	0.331	0.700	NA	0.389	0.145	0.135
		**<25**		2.918 (1.01-4.41)	4.756 (1.011-8.290)	1.434 (0.80-5.39)		0.845 (0.63-1.44)	0.849 (0.63-2.27)	0.919 (0.75-1.34)		1.061 (0.86-1.27)	1.148 (0.86-2.94)	1.087 (0.63-2.60)
		**25-30**		3.501 (0.31-8.06)	3.927 (0.313-12.30)	2.390 (0.31-12.4)		0.695 (0.55-1.30)	0.797 (0.55-130)	0.923 (0.60-1.62)		1.265 (0.78-2.14)	1.365 (0.78-2.26)	1.534 (0.70-3.24)
		**>30**		3.004 (0.31-7.41)	3.004 (0.31-7.41)	2.044 (0.31-5.07)		0.754 (0.28-1.21)	0.754 (0.28-1.21)	1.057 (0.49-8.23)		1.043 (0.45-1.74)	1.043 (0.45-1.74)	1.441 (0.79-3.84)
	continuous	***p***	0.556	0.798	0.242	0.500	0.444	0.500	0.163	0.802	0.906	0.098	**0.047**	**0.068**
		**r**	0.286	0.058	-0.224	0.102	-0.357	-0.152	-0.266	-0.038	0.071	-0.362	**-0.372**	**0.272**
**W/H ratio**	*continuous*	***p***	>0.999	0.166	0.486	0.529	>0.999	0.715	0.440	0.461	>0.999	0.366	0.620	0.960
		**r**	-0.500	0.332	0.157	0.100	0.500	-0.090	-0.174	0.117	0.500	0.220	0.112	0.008
	categorical	***p***	NA	0.599	0.853	0.976	NA	0.656	0.988	0.101	NA	0.626	0.33	0.777
**Metabolic syndrome**		**ab**		3.405 (0.31-8.06)	4.167 (0.31-12.30)	2.18 (1.02-4.36)		0.807 (0.59-1.30)	0.799 (0.29-2.27)	1.148 (0.75-2.95)		0.735 (0.29-1.44)	1.15 (0.66-2.94)	1.125 (0.63-3.01)
		**pr**		0.807 (0.59-1.30)	3.419 (2.23-7.41)	2.23 (0.31-12.43)		1.044 (0.45-1.74)	0.807 (0.58-1.30)	0.916 (0.49-8.23)		1.044 (0.45-1.74)	1.044 (0.45-1.74)	1.41 (0.70-3.84)
**Central obesity**	*categorical*	***p***	NA	0.797	0.136	0.342	NA	0.637	0.472	0.783	NA	0.557	0.555	**0.039**
		**ab**		3.922 (0.31-7.19)	5.413 (0.31-12.30)	1.398 (1.02-3.96)		0.704 (0.61-0.99)	0.822 (0.61-2.27)	1.034 (0.75-1.34)		1.016 (0.78-1.59)	1.148 (0.78-2.94)	**1.049** (0.63-1.11)
		**pr**		3.08 (0.31-8.06)	3.075 (0.31-8.06)	2.238 (0.31-12.43)		0.735 (0.28-1.30)	0.735 (0.28-1.30)	0.932 (0.49-8.23)		1.144 (0.45-2.14)	1.144 (0.45-2.14)	**1.441** (0.70-3.84)
**Family history**	*categorical*	***p***	NA	0.074	0.928	0.942	NA	0.450	0.505	0.350	NA	0.545	0.189	0.613
		**ab**		2.148 (0.31-4.66)	4.050 (0.31-12.3)	2.364 (0.31-4.21)		0.739 (0.55-1.04)	0.754 (0.51-2.27)	1.133 (0.54-2.95)		1.116 (0.76-2.14)	1.164 (0.76-2.94)	1.360 (1.01-3.24)
		**pr**		3.922 (0.71-8.06)	3.922 (0.71-8.06)	2.113 (0.31-12.43)		0.735 (0.29-1.44)	0.735 (0.29-1.44)	0.926 (0.49-8.23)		1.070 (0.45-1.95)	1.070 (0.45-1.95)	1.285 (0.63-3.84)
**Hypertension**	*categorical*	***p***	NA	0.263	0.527	0.322	NA	0.638	0.634	0.207	NA	0.652	0.429	0.831
		**ab**		3.158 (0.31-7.19)	3.922 (0.33-12.30)	1.738 (0.31-5.39)		0.719 (0.28-1.44)	0.735 (0.28-2.27)	1.057 (0.49-2.46)		1.088 (0.66-2.14)	1.128 (0.66-2.94)	1.410 (0.70-3.84)
		**pr**		5.557 (0.31-8.06)	5.557 (0.31-8.06)	2.398 (0.31-12.43)		0.773 (0.68-1.06)	0.773 (0.68-1.06)	0.879 (0.53-8.23)		1.018 (0.45-1.95)	1.018 (0.45-1.95)	
**Hyperlipidemia**	*categorical*	***p***	NA	0.334	0.913	0.515	NA	0.766	0.676	**0.028**	NA	0.266	0.132	0.225
		**ab**		2.839 (0.31-8.06)	3.922 (0.31-12.30)	1.692 (1.02-4.19)		0.72 (0.29-1.44)	0.735 (0.29-26.27)	**1.148** (0.75-2.95)		1.152 (0.66-1.95)	1.159 (0.66-2.94)	1.109 (0.63-3.01)
		**pr**		3.927 (1.75-7.41)	3.927 (1.75-7.41)	2.238 (0.31-12.43)		0.800 (0.55-1.30)	0.800 (0.55-1.30)	**0.879** (0.49-8.23)		1.016 (0.45-2.14)	1.016 (0.45-2.14)	1.44 (0.70-3.84)
**HbA1c (%)**	*categorical*	***p***	NA	NA	NA	0.985	NA	NA	NA	**0.066**	NA	NA	NA	0.348
		**<7%**				2.044 (0.31-12.43)				**0.834**				1.306 (0.63-3.55)
		**>7%**				2.480 (0.31-4.62)				**1.150**				1.449 (0.89-3.84)
	continuous	***p***	NA	0.547	0.928	0.769	NA	0.527	0.914	**0.042**	NA	0.964	0.857	0.517
		**r**		0.152	-0.022	-0.047		0.160	0.027	**0.315**		-0.012	0.044	0.103
**Glucose (mg/dl)**	*categorical*	***p***	NA	NA	NA	0.206	NA	NA	NA	0.971	NA	NA	NA	0.712
		**<130**				2.044 (0.31-12.43)				1.057 (0.49-3.03)				1.306 (0.79-3.51)
		**>130**				3.321 (0.31-5.39)				0.932 (0.60-8.23)				1.310 (0.70-3.84)
	continuous	***p***	0.983	0.146	**0.085**	0.103	0.511	0.735	0.797	0.758	0.672	0.391	**0.049**	0.787
		**r**	0.029	-0.329	**-0.338**	0.249	0.348	0.079	-0.052	0.048	-0.202	0.198	**0.810**	0.042
**Insulin (μU/ml)**	*continuous*	***p***	0.136	0.708	0.226	0.553	0.297	0.876	0.498	**0.021**	0.136	0.729	0.236	**0.019**
		**r**	0.714	-0.087	-0.222	-0.098	-0.543	0.036	-0.136	**0.368**	-0.714	-0.081	-0.236	**0.374**
**Cholesterol total (mg/dl)**	*continuous*	***p***	0.333	0.349	0.597	0.696	0.750	0.165	0.220	0.661	>0.999	0.144	0.286	0.087
		**r**	0.800	0.235	0.119	0.074	0.400	-0.342	-0.272	-0.083	0.000	-0.359	-0.238	-0.318
**HDL (mg/dl)**	*continuous*	***p***	>0.999	0.222	0.734	0.424	0.500	**0.089**	0.201	0.525	0.167	0.903	0.883	0.885
		**r**	0.316	-0.303	-0.077	-0.154	0.633	**-0.412**	-0.281	-0.123	-0.633	0.031	-0.033	0.028
**LDL (mg/dl)**	*continuous*	***p***	0.750	0.378	0.599	0.290	0.417	0.328	0.315	0.675	0.917	0.281	0.345	0.217
		**r**	0.400	0.221	0.119	0.204	-0.600	-0.245	-0.225	-0.081	0.200	-0.269	-0.211	-0.237
**Triglycerides (mg/dl)**	*continuous*	***p***	0.917	0.484	0.778	0.564	>0.999	**0.031**	**0.069**	0.802	0.750	0.404	0.820	0.303
		**r**	0.200	0.177	-0.064	-0.110	0.000	**0.509**	**0.395**	0.048	0.400	-0.210	-0.051	-0.195

Variable	Type		*CDKN2A* tv3				*CDKN2A* tv4				*IGF2BP2* tv7			
			CT_{RF-}	CT_{RF+}	CT	T2D	CT_{RF-}	CT_{RF+}	CT	T2D	CT_{RF-}	CT_{RF+}	CT	T2D
**BMI**	*ordinal*	***p***	NA	0.157	0.195	0.940	NA	**0.013**	**0.002**	NS	NA	0.461	0.777	**0.034**
		**<25**		0.057 (0.01-0.82)	0.066 (0.01-2.08)	0.062 (0.03-0.61)		**0.455** (0.36-1)	**0.555** (0.36-1)			3.462 (2.38-11.91)	3.693 (2.38-11.91)	**2.360** (1.03-9.91)
		**25-30**		0.048 (0.01-0.46)	0.043 (0.01-0.93)	0.080 (0.01-1.44)		**0.730** (0.43-1.49)	**0.740** (0.43-2.6)			4.035 (0.52-5.60)	4.043 (0.52-6.97)	**4.558** (1.64-10.3)
		**>30**		0.028 (0.01-0.43)	0.028 (0.01-0.43)	0.044 (0.01-3.48)		**1.100** (0.72-1.48)	**1.100** (0.72-1.48)			4.250 (2.31-7.64)	4.250 (2.31-7.64)	**4.746** (3.13-14.14)
	continuous	***p***	0.444	0.105	0.121	0.985	0.236	**0.037**	**0.008**	0.269	0.964	0.603	0.688	**0.017**
		**r**	0.357	-0.355	-0.294	0.003	-0.536	**0.447**	**0.482**	0.166	0.036	0.118	0.078	**0.351**
**W/H ratio**	*continuous*	***p***	>0.999	0.593	0.428	0.775	>0.999	**0.010**	**0.002**	0.200	>0.999	0.977	0.838	0.529
		**r**	0.500	-0.131	-0.178	-0.045	-0.500	**0.575**	**0.623**	0.203	-0.500	-0.007	-0.046	0.099
	categorical	***p***	NA	0.933	0.948	NS	NA	0.248	NS	0.513	NA	0.933	0.139	0.759
**Metabolic syndrome**		**ab**		0.039 (0.01-0.82)	0.039 (0.01-2.08)	0.438 (0.02-2.18)		0.800 (0.36-1.49)	0.705 (0.36-2.6)	0.997 (0.67-1.48)		3.57 (0.52-11.9)	3.922 (0.52-11.91)	4.942 (1.03-14.14)
		**pr**		0.034 (0.01-0.46)	0.034 (0.01-0.46)	0.052 (0.01-3.48)		1.05 (0.72-1.32)	1.045 (0.72-1.32)	0.90 (0.29-3.3)		4.02 (2.31-4.61)	4.016 (2.31-4.61)	4.471 (1.64-13.50)
**Central obesity**	*categorical*	***p***	NA	0.857	0.902	0.898	NA	0.136	**0.061**	0.699	NA	0.428	0.674	0.474
		**ab**		0.033 (0.01-0.16)	0.039 (0.01-2.08)	0.080 (0.03-0.51)		0.540 (0.37-1.49)	**0.644** (0.36-2.6)	0.966 (0.74-1.19)		3.35 (0.52-11.91)	3.693 (0.52-11.91)	3.585 (1.54-9.91)
		**pr**		0.039 (0.01-0.46)	0.04 (0.01-0.46)	0.069 (0.01-3.48)		0.990 (0.43-1.48)	**0.990** (0.43-1.48)	0.900 (0.29-3.30)		4.043 (2.31-7.64)	4.043 (2.31-7.64)	4.500 (1.03-14.14)
**Family history**	*categorical*	***p***	NA	0.751	0.191	0.219	NA	0.317	0.721	0.882	NA	0.238	0.400	0.316
		**ab**		0.036 (0.01-0.43)	0.024 (0.01-2.08)	0.275 (0.01-2.18)		0.985 (0.43-1.49)	0.800 (0.36-2.60)	0.938 (0.29-1.56)		4.367 (0.52-11.91)	3.908 (0.52-12.14)	4.989 (2.15-14.14)
		**pr**		0.039 (0.01-0.82)	0.039 (0.01-0.82)	0.057 (0.01-3.48)		0.740 (0.36-1.32)	0.740 (0.36-1.32)	0.910 (0.43-3.30)		3.891 (2.31-7.62)	3.891 (2.31-7.62)	4.159 (1.03-13.5)
**Hypertension**	*categorical*	***p***	NA	0.869	0.662	0.215	NA	0.156	0.156	NS	NA	0.856	0.905	0.434
		**ab**		0.039 (0.01-0.82)	0.029 (0.01-2.08)	0.082 (0.01-1.68)		0.770 (0.36-1.49)	0.720 (0.36-2.60)	0.860 (0.43-1.71)		3.940 (0.52-11.91)	3.898 (0.52-12.14)	4.500 (1.03-9.91)
		**pr**		0.030 (0.01-0.452)	0.030 (0.01-0.452)	2.398 (0.31-12.43)		1.100 (0.56-1.48)	1.100 (0.56-1.48)	0.94 (0.29-3.3)		4.609 (2.31-7.64)	4.609 (2.31-7.64)	4.184 (1.64-14.14)
**Hyperlipidemia**	*categorical*	***p***	NA	0.766	0.326	0.101	NA	0.707	0.952	0.128	NA	0.739	0.786	0.959
		**ab**		0.036 (0.01-0.82)	0.029 (0.01-2.08)	0.506 (0.02-2.18)		0.91 (0.36-1.49)	0.72 (0.36-2.6)	1.090 (0.67-1.48)		3.96 (0.52-11.91)	3.898 (0.52-12.14)	4.942 (1.54-14.14)
		**pr**		2.561 (1.55-4.08)	2.561 (1.55-4.08)	2.238 (0.31-12.43)		0.800 (0.43-1.10)	0.800 (0.43-1.10)	0.870 (0.29-3.30)		3.989 (2.31-4.61)	3.989 (2.31-4.61)	4.500 (1.03-13.50)
**HbA1c (%)**	*categorical*	***p***	NA	NA	NA	0.601	NA	NA	NA	0.322	NA	NA	NA	0.419
		**<7%**				0.062 (0.01-2.46)				0.880 (0.29-3.30)				4.184 (1.03-14.14)
		**>7%**				0.078 (0.01-3.48)				1.090 (0.52-2.87)				4.616 (1.64-13.5)
	continuous	***p***	NA	0.300	0.782	0.654	NA	0.074	0.170	0.069	NA	0.761	0.469	0.909
		**r**		0.259	0.068	0.071		0.432	0.329	0.284		-0.077	-0.177	-0.018
**Glucose (mg/dl)**	*categorical*	***p***	NA	NA	NA	0.827	NA	NA	NA	**0.073**	NA	NA	NA	0.249
		**<130**				0.069 (0.01-2.46)				**0.980 (0.29-3.30)**				4.447 (1.64-14.14)
		**>130**				0.082 (0.01-3.48)				**0.850 (0.44-2.87)**				6.636 (2.51-13.5)
	continuous	***p***	**0.078**	0.801	0.823	0.999	0.122	0.364	0.619	**0.083**	0.933	**0.017**	**0.086**	0.314
		**r**	**0.783**	-0.058	-0.045	-0.0002	-0.696	0.209	0.100	**-0.264**	0.058	**0.513**	**0.336**	0.155
**Insulin (μU/ml)**	*continuous*	***p***	0.803	0.749	0.402	**0.008**	0.658	0.407	0.378	0.282	0.356	**0.026**	**0.005**	0.241
		**r**	-0.143	0.074	0.168	**0.419**	0.257	0.191	0.177	0.177	-0.486	**0.486**	**0.522**	0.192
**Cholesterol total (mg/dl)**	*continuous*	***p***	**0.083**	0.212	0.109	0.786	0.333	0.946	0.900	0.170	0.417	0.553	0.304	0.204
		**r**	**1.000**	-0.309	-0.351	0.052	-0.800	0.017	0.034	-0.257	0.600	-0.149	-0.229	0.239
**HDL (mg/dl)**	*continuous*	***p***	>0.999	0.668	0.542	0.512	>0.999	**0.022**	**0.003**	0.225	0.500	0.692	0.624	0.351
		**r**	0.316	0.109	0.137	-0.127	0.316	**-0.534**	**-0.609**	-0.232	-0.316	-0.100	-0.111	-0.180
**LDL (mg/dl)**	*continuous*	***p***	0.750	0.285	0.245	0.973	0.333	0.646	0.382	0.441	0.750	0.394	0.411	0.317
		**r**	0.400	-0.266	-0.259	-0.007	-0.800	0.116	0.200	-0.149	0.400	-0.214	-0.145	0.193
**Triglycerides (mg/dl)**	*continuous*	***p***	0.333	0.887	0.684	0.115	0.083	0.176	**0.037**	0.734	0.333	0.433	0.368	**0.052**
		**r**	0.800	0.036	-0.092	0.293	-1.000	0.337	**0.447**	0.065	0.800	-0.197	-0.202	**0.358**

Variable	Type		*KCNQ1 tv1*				*THADA tv5*				*TSPAN8*			
			CT_{RF-}	CT_{RF+}	CT	T2D	CT_{RF-}	CT_{RF+}	CT	T2D	CT_{RF-}	CT_{RF+}	CT	T2D
**BMI**	*ordinal*	***p***	NA	0.243	**0.052**	0.129	NA	0.667	0.627	0.932	NA	0.580	0.418	0.080
		**<25**		8.795 (2.45-15.16)	**11.860** (5.70-16.62)	11.80 (3.73-12.56)		2.122 (0.89-14.19)	2.122 (0.89-35.83)	5.312 (0.72-19.49)		0.178 (0.09-0.73)	0.147 (0.09-1.13)	0.226 (0.08-0.50)
		**25-30**		8.795 (2.45-15.16)	**8.951** (2.45-15.16)	9.024 (3.14-16.93)		3.718 (0.68-16.01)	3.304 (0.68-16.47)	4.029 (1.10-31.6)		0.189 (0.07-0.49)	0.197 (0.07-0.63)	0.221 (0.04-0.65)
		**>30**		7.221 (4.94-16.34)	**7.221** (4.94-16.34)	6.763 (2.88-12.2)		2.209 (0.60-12.85)	2.209 (0.60-12.85)	2.756 (0.90-120.7)		0.193 (0.12-0.83)	0.193 (0.12-0.83)	0.246 (0.07-2.12)
	continuous	***p***	0.662	0.425	0.166	0.213	0.556	0.398	0.482	0.762	0.267	0.712	0.660	0.181
		**r**	-0.214	-0.179	-0.264	-0.187	0.286	-0.190	-0.136	-0.046	0.500	0.084	0.085	0.201
**W/H ratio**	*continuous*	***p***	0.333	0.932	0.948	0.493	>0.999	0.571	0.478	0.627	>0.99	0.623	0.838	0.466
		**r**	-1.000	0.021	-0.015	-0.109	0.500	-0.139	-0.160	0.077	0.500	0.121	0.046	0.116
	categorical	***p***	NA	0.758	0.868	0.928	NA	0.626	0.790	0.196	NA	0.674	0.714	0.865
**Metabolic syndrome**		**ab**		8.95 (2.45-16.34)	8.256 (2.45-16.62)	8.444 (3.14-16.5)		2.304 (0.60-16.1)	2.466 (0.60-35.83)	10.02 (0.72-37.17)		0.189 (0.07-0.83)	0.193 (0.07-1.13)	0.241 (0.08-0.53)
		**pr**		8.906 (7.53-13.24)	8.906 (7.53-13.24)	8.469 (2.88-16.93)	NA	2.211 (1.13-14.23)	2.211 (1.13-14.23)	3.186 (1.10-120.7)	NA	0.177 (0.12-0.36)	0.177 (0.12-0.36)	0.225 (0.04-2.12)
**Central obesity**	*categorical*	***p***	NA	0.162	**0.046**	0.256	NA	0.228	0.583	0.646	NA	**0.088**	0.267	0.367
		**ab**		10.29 (2.45-16.3)	**10.140** (2.45-16.62)	11.97 (4.65-16.50)		1.616 (0.69-6.71)	1.960 (0.69-35.83)	5.496 (2.51-10.29)		**0.162** (0.07-0.25)	0.171 (0.07-1.13)	0.217 (0.09-0.24)
		**pr**		8.39 (4.94-16.3)	**8.387** (4.94-16.34)	8.444 (2.88-16.93)		2.538 (0.60-16.01)	2.538 (0.60-16.01)	3.396 (0.72-120.7)		**0.197** (0.09-0.83)	0.197 (0.09-0.83)	0.246 (0.04-2.12)
**Family history**	*categorical*	***p***	NA	0.968	0.385	0.803	NA	0.945	0.591	0.582	NA	0.098	**0.004**	0.493
		**ab**		9.315 (2.45-16.32)	9.748 (2.44-16.62)	8.244 (4.65-16.5)		2.094 (0.69-12.85)	2.625 (0.69-35.83)	5.266 (0.91-37.2)		0.152 (0.07-0.83)	**0.111** (0.03-1.13)	0.265 (0.07-0.82)
		**pr**		8.951 (4.94-16.34)	8.951 (4.94-16.34)	8.744 (2.88-16.9)		2.538 (0.60-16.01)	2.538 (0.60-16.01)	3.452 (0.72-120.7)		0.198 (0.09-0.73)	**0.198** (0.09-0.73)	0.221 (0.04-2.12)
**Hypertension**	*categorical*	***p***	NA	0.339	0.195	0.378	NA	0.967	0.666	0.570	NA	0.257	**0.078**	0.799
		**ab**		9.164 (2.45-16.34)	9.357 (2.45-16.62)	9.765 (3.14-16.9)		2.421 (0.60-14.23)	2.625 (0.60-35.83)	5.312 (0.72-33.2)		0.167 (0.07-0.83)	**0.133** (0.02-1.13)	0.241 (0.08-0.66)
		**pr**		7.526 (4.96-13.24)	7.526 (4.96-13.24)	8.444 (2.88-16.21)		1.881 (1.13-16.01)	1.881 (1.13-16.01)	3.186 (0.91-120.7)		0.197 (0.19-0.39)	**0.197** (0.19-0.39)	0.213 (0.04-2.12)
**Hyperlipidemia**	*categorical*	***p***	NA	0.089	0.226	0.294	NA	0.669	0.999	**0.028**	NS	0.36	0.65	0.283
		**ab**		8.115 (2.45-16.34)	8.951 (2.45-16.62)	6.06 (3.14-16.5)		1.882 (0.60-16.01)	2.538 (0.6-35.83)	**10.290** (0.9-37.17)		0.193 (0.07-0.83)	0.143 (0.03-1.13)	0.357 (0.09-0.53)
		**pr**		9.992 (7.53-16.32)	9.992 (7.53-16.32)	9.020 (2.88-16.93)		2.629 (1.13-14.23)	2.629 (1.13-14.23)	**3.039** (0.7-12.07)		0.162 (0.09-0.36)	0.162 (0.09-0.36)	0.217 (0.04-2.12)
**HbA1c (%)**	*categorical*	***p***	NA	NA	NA	0.566	NA	NA	NA	0.638	NA	NA	NA	0.955
		**<7%**				8.045 (3.14-16.5)				3.039 (0.72-57.4)				0.213 (0.07-2.12)
		**>7%**				9.765 (3.62-16.9)				3.530 (1.10-120.7)				0.226 (0.04-2.05)
	continuous	***p***	NA	**0.084**	**0.038**	0.683	NA	0.423	0.937	0.518	NA	0.292	0.634	0.766
		**r**		**-0.419**	**-0.480**	-0.065		0.201	0.019	0.103		0.263	0.117	0.047
**Glucose (mg/dl)**	*categorical*	***p***	NA	NA	NA	0.221	NA	NA	NA	0.195	NA	NA	NA	0.952
		**<130**				6.763 (2.88-16.5)				2.756 (0.91-57.41)				0.241 (0.07-2.12)
		**>130**				9.020 (3.62-16.9)				4.529 (1.31-120.7)				0.247 (0.04-2.05)
	continuous	***p***	0.983	0.520	0.322	0.134	0.206	0.339	0.753	0.356	0.672	0.148	0.163	0.728
		**r**	0.000	-0.149	-0.198	0.230	0.609	0.220	0.064	0.143	0.232	0.327	0.417	-0.054
**Insulin (μU/ml)**	*continuous*	***p***	0.658	0.379	0.498	**0.067**	0.497	0.404	0.290	**0.002**	>0.999	0.141	0.161	**0.039**
		**r**	0.257	-0.203	-0.136	**-0.296**	-0.371	0.192	0.211	**0.476**	-0.029	0.333	0.278	**0.341**
**Cholesterol total (mg/dl)**	*continuous*	***p***	0.333	0.534	0.237	0.144	0.333	0.091	**0.061**	0.823	0.333	0.369	0.395	0.732
		**r**	-0.800	-0.157	-0.263	0.274	0.800	-0.410	**-0.405**	-0.043	0.800	-0.225	-0.191	-0.065
**HDL (mg/dl)**	*continuous*	***p***	0.500	0.239	0.622	0.265	0.500	0.601	0.491	0.538	0.500	0.708	0.469	0.359
		**r**	-0.316	-0.292	-0.114	0.214	-0.316	-0.132	-0.155	-0.119	-0.316	-0.095	-0.163	-0.177
**LDL (mg/dl)**	*continuous*	***p***	>0.999	0.139	**0.028**	0.549	0.333	0.069	0.123	0.695	0.333	0.681	0.923	0.937
		**r**	0.000	-0.362	**-0.468**	0.116	0.800	-0.438	-0.339	-0.076	0.800	-0.104	0.022	0.015
**Triglycerides (mg/dl)**	*continuous*	***p***	0.417	0.938	0.309	0.231	0.083	0.372	0.443	0.116	0.083	0.639	0.394	0.109
		**r**	-0.600	-0.020	-0.228	-0.225	1.000	0.224	0.172	0.293	1.000	0.119	0.191	0.300

NA: not applicable; NS: not significant

CT_{TOTAL}: the total cohort of controls; CT_{RF-}: controls with risk factors for T2D; CT_{RF+}: controls without risk factors for T2D; T2D: patients

Supplemental Table 4. eQTLS linked to T2D-related SNPs on the differentially expressed genes, in whole blood cells and/or T2D-target tissues. Data were obtained from the Blood eQTL browser and the GTEx portal. From the Blood eQTL database, data on all the cis- or trans- eQTLs associated with T2D-related SNPs in the genes-of-interest detected in human blood samples were obtained. Significance p-values, z-scores, false discovery rates (FDR) and the names of the affected genes are reported for all eQTLs (cut-off: FDR=0.05). From the GTEx portal, data on eQTLs associated with T2D-related SNPs in the genes-of-interest detected in whole blood and T2D-target tissues are reported. Significance p-value, effect size (beta-score), the name of the affected genes in certain tissue together with the number of samples tested in the GTEx project so far, are reported. Moreover, data from cross-tissue meta-analysis for each eQTL are provided: The tissue is predicted to have an eQTL effect if multi-tissue posterior probability (m-value) is >0.9.

Blood eQTL browser

Cis/Trans eQTL	P-value	SNP	SNP Chr.	SNP Chr. Position	Probe	Probe Chr.	Probe Chr. position	SNP Alleles	Minor Allele	Z-score	Gene Symbol	Full Gene Name	FDR
CAPN10													
rs3792267													
No records found													
rs5030952													
No records found													
CDKN2A													
rs2383208													
trans	2.5e-6	rs2383208	9	22122076	3800753	7	21908064	G/A	G	4.71	*CDCA7L*	Cell division cycle associated 7 like	0.17
trans	4.7e-6	rs2383208	9	22122076	520521	1	1332497	G/A	G	-4.58	*MRPL20*	Mitochondrial ribosomal protein L20	0.26

GTEx Portal

Gencode Id	Gene Symbol	Full Gene Name	Variant Id	SNP Id	P-Value	Sample No	Beta	m-value	Tissue
CAPN10									
rs3792267									
No records found									
rs5030952									
ENSG00000178623.7	*GPR35*	G protein-coupled receptor 35	2_241542703_C_T_b37	rs5030952	2.8e-9	369	0.415	1.00	Whole Blood
ENSG00000142330.15	*CAPN10*	Caplain-10	2_241542703_C_T_b37	rs5030952	0.0000094	491	0.235	1.00	Muscle - Skeletal
CDKN2A									
rs2383208									
No records found									

trans	9.6e-6	rs2383208	9	22122076	3840730	14	72673597	G/A	G	-4.43	*PSEN1*	Presenilin 1	0.39	
rs10811661														
trans	2.2e-6	rs10811661	9	22124094	3800753	7	21908064	T/C	C	4.74	*CDCA7L*	Cell division cycle associated 7 like	0.16	*No records found*
trans	5.5e-6	rs10811661	9	22124094	3840730	14	72673597	T/C	C	-4.54	*PSEN1*	Presenilin 1	0.29	
trans	5.9e-6	rs10811661	9	22124094	520521	1	1332497	T/C	C	-4.53	*MRPL20*	Mitochondrial ribosomal protein L20	0.30	
rs564398														
trans	5.5e-6	rs564398	9	22019547	1940707	7	65913136	T/C	C	4.55	*RABGEF1*	RAB guanine nucleotide exchange factor 1	0.29	*No records found*
trans	8.5e-6	rs564398	9	22019547	3890681	3	127208012	T/C	C	-4.45	*SLC41A3*	Solute carrier family 41 member 3	0.37	
rs1333051														
trans	6.2e-7	rs1333051	9	22126489	3800753	7	21908064	T/A	T	4.98	*CDCA7L*	Cell division cycle associated 7 like	0.06	*No records found*
rs7020996														

trans	1.4e-5	rs7020996	9	22119579	4760619	5	37871321	T/C	T	-4.34	*GDNF*	Glial cell-derived neurotrophic factor	0.48	*No records found*
rs10965250														
trans	5.6e-6	rs10965250	9	22123284	3800753	7	21908064	G/A	A	4.54	*CDCA7L*	Cell division cycle associated 7 like	0.29	*No records found*
trans	9.4e-6	rs10965250	9	22123284	520521	1	1332497	G/A	A	-4.43	*MRPL20*	Mitochondrial ribosomal protein L20	0.39	
rs2383208														
trans	2.5e-6	rs2383208	9	22122076	3800753	7	21908064	G/A	G	4.71	*CDCA7L*	Cell division cycle associated 7 like	0.17	*No records found*
trans	4.7e-6	rs2383208	9	22122076	520521	1	1332497	G/A	G	-4.58	*MRPL20*	Mitochondrial ribosomal protein L20	0.26	
trans	9.6e-6	rs2383208	9	22122076	3840730	14	72673597	G/A	G	-4.43	*PSEN1*	Presenilin 1	0.39	
IGF2BP2														
rs4402960														
cis	4.0e-9	rs4402960	3	186994381	2940349	3	186830982	T/G	T	5.88	*SENP2*	Sentrin-specific protease 2	0.00	*No records found*

trans	1.3e-5	rs4402960	3	186994381	360692	23	145963580	T/G	T	-4.36	-		0.46	
**rs1374910**														
cis	1.2e-5	rs1374910	3	187014355	2940349	3	186830982	C/T	T	4.38	*SENP2*	Sentrin-specific protease 2	0.01	*No records found*
trans	7.3e-6	rs1374910	3	187014355	4570181	13	113555142	C/T	T	-4.49	*GAS6*	Growth arrest-specific 6	0.34	
**rs1470579**														
cis	9.6e-9	rs1470579	3	187011774	2940349	3	186830982	C/A	C	5.74	*SENP2*	Sentrin-specific protease 2	0.00	*No records found*
trans	1.5e-5	rs1470579	3	187011774	360692	23	145963580	C/A	C	-4.33	-		0.49	
**rs138306797**														
No records found														*No records found*
**rs6769511**														
cis	1.3e-8	rs6769511	3	187012984	2940349	3	186830982	T/C	C	5.69	*SENP2*	Sentrin-specific protease 2	0.00	*No records found*
**rs11927381**														
No records found														*No records found*
KCNQ1														
**rs2237892**														
No records found														*No records found*
**rs163182**														

No records found													*No records found*
rs2237895													
No records found													*No records found*
rs2237897													
trans	6.4e-6	rs2237897	11	2815122	3290465	2	165954646	C/T	T	-4.51	*SCN2A2*	Sodium voltage-gated channel alpha subunit 2	0.32	*No records found*
trans	8.3e-6	rs2237897	11	2815122	6860048	1	7835978	C/T	T	-4.46	*UTS2*	Urotensin 2	0.37	
rs231362													
No records found													*No records found*
rs2283228													
No records found													*No records found*
rs8181588													
No records found													*No records found*
rs163184													
No records found													*No records found*
rs2237896													
No records found													*No records found*
rs117601636													
No records found													*No records found*
THADA													
rs7578597													
No records found													*No records found*
TSPAN8													
rs7961581													
No records found													*No records found*
rs4760790													

No records found	ENSG00000258053.1	*CTD-2021H9.3*		12_71634794_A_G_b37	rs4760790	0.000021	491	-0.230	1.00	Muscle - Skeletal
rs1495377										
No records found	ENSG00000258053.1	*CTD-2021H9.3*		12_71577101_G_C_b37	rs1495377	1.1e-10	491	-0.296	1.00	Muscle - Skeletal

Supplemental Table 5. Data on cis-eQTLs acting upon *CAPN10* expression in human whole blood cells, adipose tissue cells and skeletal muscle (light-blue rows), obtained from the GTEx portal (5/3/2018). For the identification of significant eGENES, the GTEx project applied a permutation-based approach in which nominal p-values were generated for each variant-gene pair by testing the alternative hypothesis that the slope of a linear regression model between genotype and expression deviates from 0, beta distribution-adjusted empirical p-values were used to calculate q-values, and a false discovery rate (FDR) threshold of ≤0.05 was applied. The effect size (Allelic Fold-Change) of cis-acting genetic variation on CAPN10 mRNA levels in each tissue is also reported. Moreover, for each one of the tissues-of-interest where *CAPN10* is identified as an eGENE, all the related eQTLs are listed. For each one, of these eQTLs, the corresponding variant/SNP, the significance p-value, the gene and its transcript variant(s), where the SNP harbors, as well as any disease/trait associated are reported.

Tissue	Gencode Id	Gene Symbol	Nominal P-Value	Empirical P-Value	q-value		Allelic Fold-Change
**Adipose - Subcutaneous**	ENSG00000142330.15	***CAPN10***	9,23E-07	7,46E-02	0.00000122596		-0.204679
		**Variant ID**	**SNP ID**	**P-Value**	**Gene (tvor non-coding region) bearing the SNP**	**Full name of the gene**	**Known disease/trait association (GWAS catalog/SNPedia)**
		2_241526862_C_T_b37	rs67448739	9.2e-11	*CAPN10 tv1, tv3*	Calpain-10	
		2_241527282_G_A_b37	rs67940783	9.9e-11	*CAPN10 tv1, tv3*	Calpain-10	
		2_241537047_C_T_b37	rs56960757	1.3e-10	*CAPN10 tv1, tv3*	Calpain-10	
		2_241538061_T_C_b37	rs55878652	1.3e-10	*CAPN10 tv1, tv3*	Calpain-10	
		2_241540110_A_G_b37	rs12616820	1.4e-10	non-coding region of *CAPN10*	Calpain-10	
		2_241526259_T_A_b37	rs55650580	1.6e-10	*CAPN10 tv1, tv3*	Calpain-10	
		2_241526053_A_T_b37	rs56352308	2.2e-10	*CAPN10 tv1, tv3*	Calpain-10	
		2_241526167_G_A_b37	rs56244291	2.2e-10	*CAPN10 tv1, tv3*	Calpain-10	
		2_241527462_G_T_b37	rs7596933	2.2e-10	*CAPN10 tv1, tv3*	Calpain-10	
		2_241528011_G_A_b37	rs12619657	2.2e-10	*CAPN10 tv1, tv3*	Calpain-10	
		2_241528927_G_A_b37	rs55893551	2.2e-10	*CAPN10 tv1, tv3*	Calpain-10	
		2_241528968_G_C_b37	rs55976923	2.2e-10	*CAPN10 tv1, tv3*	Calpain-10	
		2_241530616_A_T_b37	rs2975757	2.2e-10	*CAPN10 tv1, tv3*	Calpain-10	
		2_241531163_T_C_b37	rs2975760	2.2e-10	*CAPN10 tv1, tv3*	Calpain-10	
		2_241531479_A_G_b37	rs3792269	2.2e-10	*CAPN10 tv1, tv3*	Calpain-10	
		2_241532559_G_C_b37	rs41266975	2.2e-10	*CAPN10 tv1, tv3*	Calpain-10	
		2_241532620_C_A_b37	rs56783920	2.2e-10	*CAPN10 tv1, tv3*	Calpain-10	
		2_241533030_T_C_b37	rs3792273	2.2e-10	*CAPN10 tv1, tv3*	Calpain-10	
		2_241534163_G_A_b37	rs17846975	2.2e-10	*CAPN10 tv1, tv3*	Calpain-10	
		2_241536819_C_A_b37	rs3828345	2.2e-10	*CAPN10 tv1, tv3*	Calpain-10	
		2_241537569_T_C_b37	rs17846981	2.2e-10	*CAPN10 tv1, tv3*	Calpain-10	
		2_241538924_C_G_b37	rs67211173	2.2e-10	*CAPN10 tv1, tv3*	Calpain-10	
		2_241539003_A_G_b37	rs2953168	2.2e-10	*CAPN10 tv1, tv3*	Calpain-10	
		2_241539478_C_T_b37	rs57337179	2.4e-10	non-coding region of *CAPN10*	Calpain-10	
		2_241536692_G_A_b37	rs3792275	2.5e-10	*CAPN10 tv1, tv3*	Calpain-10	
		2_241536126_A_G_b37	rs7607759	2.5e-10	*CAPN10 tv1, tv3*	Calpain-10	
		2_241528389_G_A_b37	rs55999124	3.3e-10	*CAPN10 tv1, tv3*	Calpain-10	
		2_241539683_T_C_b37	rs67745756	4.0e-10	non-coding region of *CAPN10*	Calpain-10	
		2_241536053_C_T_b37	rs2953171	4.5e-10	*CAPN10 tv1, tv3*	Calpain-10	
		2_241577986_GA_G_b37	rs35638765	5.1e-9	non-coding region	Calpain-10	
		2_241540229_G_T_b37	rs60001881	1.1e-8	non-coding region of *CAPN10*	Calpain-10	
		2_241556826_A_C_b37	rs17846976	1.6e-8	*GPR35 tv2, tv3*	G protein-coupled receptor 35	
		2_241516094_G_C_b37	rs34344571	1.9e-8	*RNPEPL1*	Arginyl aminopeptidase like 1	
		2_241523790_G_A_b37	rs144304188	3.5e-8	non-coding variant of *CAPN10*	Calpain-10	
		2_241539943_C_T_b37	rs74733171	4.0e-8	non-coding region of *CAPN10*	Calpain-10	

2_241541129_A_T_b37	rs73003705	4.0e-8	non-coding region		
2_241541150_G_C_b37	rs73003707	4.0e-8	non-coding region		
2_241541811_C_T_b37	rs114966520	4.0e-8	non-coding region		
2_241543594_G_A_b37	rs73003723	4.0e-8	*GPR35 tv2, tv3*	G protein-coupled receptor 35	
2_241543863_C_T_b37	rs66815376	4.0e-8	*GPR35 tv2, tv3*	G protein-coupled receptor 35	
2_241546271_T_G_b37	rs6725511	4.0e-8	*GPR35 tv2, tv3*	G protein-coupled receptor 35	
2_241552482_C_T_b37	rs73005866	4.0e-8	*GPR35 tv2, tv3*	G protein-coupled receptor 35	
2_241554018_C_T_b37	rs56086919	4.0e-8	*GPR35 tv2, tv3*	G protein-coupled receptor 35	
2_241558134_A_G_b37	rs73005895	4.0e-8	*GPR35 tv2, tv3*	G protein-coupled receptor 35	
2_241516241_C_T_b37	rs76791955	4.7e-8	*RNPEPL1*	Arginyl aminopeptidase like 1	
2_241517847_CAG_C_b37	rs3833579	4.7e-8	*RNPEPL1*	Arginyl aminopeptidase like 1	
2_241518105_G_C_b37	rs1127207	4.7e-8	*RNPEPL1*	Arginyl aminopeptidase like 1	
2_241518998_A_G_b37	rs72999942	4.7e-8	non-coding region		
2_241523664_G_A_b37	rs139537724	4.7e-8	non-coding variant of *CAPN10*	Calpain-10	
2_241524208_T_C_b37	rs7604368	4.7e-8	non-coding variant of *CAPN10*	Calpain-10	
2_241525022_G_A_b37	rs72999959	4.7e-8	non-coding variant of *CAPN10*	Calpain-10	
2_241525599_GA_G_b37	rs3833580	4.7e-8	non-coding variant of *CAPN10*	Calpain-10	
2_241525840_C_T_b37	rs56373697	4.7e-8	*CAPN10 tv1, tv3*	Calpain-10	
2_241529078_G_C_b37	rs56351903	4.7e-8	*CAPN10 tv1, tv3*	Calpain-10	
2_241531575_A_G_b37	rs3792270	4.7e-8	*CAPN10 tv1, tv3*	Calpain-10	
2_241531954_G_A_b37	rs56027088	4.7e-8	*CAPN10 tv1, tv3*	Calpain-10	
2_241533934_T_C_b37	rs17846973	4.7e-8	*CAPN10 tv1, tv3*	Calpain-10	
2_241535036_C_T_b37	rs73001804	4.7e-8	*CAPN10 tv1, tv3*	Calpain-10	
2_241539071_T_C_b37	rs73001880	4.7e-8	non-coding variant of *CAPN10*	Calpain-10	
2_241552154_C_T_b37	rs73005864	5.8e-8	*GPR35 tv2, tv3*	G protein-coupled receptor 35	
2_241579108_G_T_b37	rs4676406	1.5e-7	non-coding region of *GPR35*	G protein-coupled receptor 35	ulcerative colitis, autoimmune thyroid disease, type I diabetes mellitus, common variable immunodeficiency, chronic childhood arthritis, ankylosing spondylitis, psoriasis, celiac disease, Crohn's disease, autoimmune disease, systemic lupus erythematosus, sclerosing cholangitis
2_241553449_A_G_b37	rs56026499	3.3e-7	*GPR35 tv2, tv3*	G protein-coupled receptor 35	

		2_241584036_C_T_b37	rs12478375	3.9e-7	non-coding region		
		2_241524427_TG_T_b37	rs149599200	0.0000014	non-coding variant of *CAPN10*	Calpain-10	
		2_241530750_G_A_b37	rs2975758	0.000014	*CAPN10 tv1, tv3*	Calpain-10	
		2_241539366_T_C_b37	rs2953167	0.000014	non-coding region of *CAPN10*	Calpain-10	
		2_241539863_G_C_b37	rs2953166	0.000014	non-coding region of *CAPN10*	Calpain-10	
		2_241538229_A_G_b37	rs2975767	0.000023	*CAPN10 tv1, tv3*	Calpain-10	
		2_241621811_G_A_b37	rs376333472	0.000026	non-coding transcript of *AQP12B*	Aquaporin 12B	
		2_241534381_A_G_b37	rs2975764	0.000028	*CAPN10 tv1, tv3*	Calpain-10	
		2_241583676_A_G_b37	rs4676403	0.000030	non-coding region		
		2_241520754_T_A_b37	rs2953176	0.000034	non-coding region		
		2_241522634_G_A_b37	rs2975754	0.000034	non-coding variant of *CAPN10*	Calpain-10	
		2_241522826_GTTC_G_b37	rs10554804	0.000034	non-coding region of *CAPN10*	Calpain-10	
		2_241574401_G_A_b37	rs4676408	0.000035	non-coding region of *GPR35*	G protein-coupled receptor 35	Inflammatory bowel disease, autoimmune thyroid disease, type I diabetes mellitus, Common variable immunodeficiency, chronic childhood arthritis, ankylosing spondylitis, psoriasis, celiac disease, ulcerative colitis, Crohn's disease, autoimmune disease, systemic lupus erythematosus
**Adipose - Visceral (Omentum)**	ENSG00000142330.15	***CAPN10***	1,56E-03	0.0000864747	0.000176838		-0.188874
		**Variant ID**	**SNP ID**	**P-Value**	**Gene (tv or non-coding region) bearing the SNP**	**Full name of the gene**	**Known disease/trait association (GWAS catalog/SNPedia)**
		2_241526862_C_T_b37	rs67448739	1.6e-8	*CAPN10 tv1, tv3*	Calpain-10	
		2_241537047_C_T_b37	rs56960757	3.7e-8	*CAPN10 tv1, tv3*	Calpain-10	
		2_241536126_A_G_b37	rs7607759	5.0e-8	*CAPN10 tv1, tv3*	Calpain-10	
		2_241526259_T_A_b37	rs55650580	5.7e-8	*CAPN10 tv1, tv3*	Calpain-10	
		2_241526053_A_T_b37	rs56352308	7.5e-8	*CAPN10 tv1, tv3*	Calpain-10	
		2_241526167_G_A_b37	rs56244291	7.5e-8	*CAPN10 tv1, tv3*	Calpain-10	
		2_241527282_G_A_b37	rs67940783	7.5e-8	*CAPN10 tv1, tv3*	Calpain-10	
		2_241527462_G_T_b37	rs7596933	7.5e-8	*CAPN10 tv1, tv3*	Calpain-10	
		2_241528011_G_A_b37	rs12619657	7.5e-8	*CAPN10 tv1, tv3*	Calpain-10	
		2_241528927_G_A_b37	rs55893551	7.5e-8	*CAPN10 tv1, tv3*	Calpain-10	
		2_241528968_G_C_b37	rs55976923	7.5e-8	*CAPN10 tv1, tv3*	Calpain-10	
		2_241530616_A_T_b37	rs2975757	7.5e-8	*CAPN10 tv1, tv3*	Calpain-10	
		2_241531163_T_C_b37	rs2975760	7.5e-8	*CAPN10 tv1, tv3*	Calpain-10	
		2_241531479_A_G_b37	rs3792269	7.5e-8	*CAPN10 tv1, tv3*	Calpain-10	
		2_241532559_G_C_b37	rs41266975	7.5e-8	*CAPN10 tv1, tv3*	Calpain-10	
		2_241532620_C_A_b37	rs56783920	7.5e-8	*CAPN10 tv1, tv3*	Calpain-10	
		2_241533030_T_C_b37	rs3792273	7.5e-8	*CAPN10 tv1, tv3*	Calpain-10	

2_241534163_G_A_b37	rs17846975	7.5e-8	*CAPN10 tv1, tv3*	Calpain-10
2_241536692_G_A_b37	rs3792275	7.5e-8	*CAPN10 tv1, tv3*	Calpain-10
2_241536819_C_A_b37	rs3828345	7.5e-8	*CAPN10 tv1, tv3*	Calpain-10
2_241537569_T_C_b37	rs17846981	7.5e-8	*CAPN10 tv1, tv3*	Calpain-10
2_241538061_T_C_b37	rs55878652	7.5e-8	*CAPN10 tv1, tv3*	Calpain-10
2_241538924_C_G_b37	rs67211173	7.5e-8	*CAPN10 tv1, tv3*	Calpain-10
2_241539003_A_G_b37	rs2953168	7.5e-8	*CAPN10 tv1, tv3*	Calpain-10
2_241539478_C_T_b37	rs57337179	7.5e-8	non-coding region of *CAPN10*	Calpain-10
2_241539683_T_C_b37	rs67745756	7.5e-8	non-coding region of *CAPN10*	Calpain-10
2_241528389_G_A_b37	rs55999124	1.1e-7	*CAPN10 tv1, tv3*	Calpain-10
2_241536053_C_T_b37	rs2953171	1.5e-7	*CAPN10 tv1, tv3*	Calpain-10
2_241540110_A_G_b37	rs12616820	1.6e-7	non-coding region of *CAPN10*	Calpain-10
2_241540229_G_T_b37	rs60001881	0.0000015	non-coding region of *CAPN10*	Calpain-10
2_241552154_C_T_b37	rs73005864	0.0000020	*GPR35 tv2, tv3*	G protein-coupled receptor 35
2_241552482_C_T_b37	rs73005866	0.0000033	*GPR35 tv2, tv3*	G protein-coupled receptor 35
2_241516094_G_C_b37	rs34344571	0.0000051	*RNPEPL1*	Arginyl aminopeptidase like 1
2_241516241_C_T_b37	rs76791955	0.0000051	*RNPEPL1*	Arginyl aminopeptidase like 1
2_241517847_CAG_C_b37	rs3833579	0.0000051	*RNPEPL1*	Arginyl aminopeptidase like 1
2_241518105_G_C_b37	rs1127207	0.0000051	*RNPEPL1*	Arginyl aminopeptidase like 1
2_241518998_A_G_b37	rs72999942	0.0000051	non-coding region	
2_241523664_G_A_b37	rs139537724	0.0000051	non-coding variant of *CAPN10*	Calpain-10
2_241524208_T_C_b37	rs7604368	0.0000051	non-coding variant of *CAPN10*	Calpain-10
2_241525022_G_A_b37	rs72999959	0.0000051	non-coding variant of *CAPN10*	Calpain-10
2_241525599_GA_G_b37	rs3833580	0.0000051	non-coding variant of *CAPN10*	Calpain-10
2_241525840_C_T_b37	rs56373697	0.0000051	*CAPN10 tv1, tv3*	Calpain-10
2_241529078_G_C_b37	rs56351903	0.0000051	*CAPN10 tv1, tv3*	Calpain-10
2_241531575_A_G_b37	rs3792270	0.0000051	*CAPN10 tv1, tv3*	Calpain-10
2_241531954_G_A_b37	rs56027088	0.0000051	*CAPN10 tv1, tv3*	Calpain-10
2_241533934_T_C_b37	rs17846973	0.0000051	*CAPN10 tv1, tv3*	Calpain-10
2_241535036_C_T_b37	rs73001804	0.0000051	*CAPN10 tv1, tv3*	Calpain-10
2_241539071_T_C_b37	rs73001880	0.0000051	non-coding variant of *CAPN10*	Calpain-10
2_241539943_C_T_b37	rs74733171	0.0000051	non-coding region of *CAPN10*	Calpain-10

		2_241541129_A_T_b37	rs73003705	0.0000051	non-coding region		
		2_241541150_G_C_b37	rs73003707	0.0000051	non-coding region		
		2_241541811_C_T_b37	rs114966520	0.0000051	non-coding region		
		2_241543863_C_T_b37	rs66815376	0.0000058	*GPR35 tv2, tv3*	G protein-coupled receptor 35	
		2_241543594_G_A_b37	rs73003723	0.0000093	*GPR35 tv2, tv3*	G protein-coupled receptor 35	
		2_241546271_T_G_b37	rs6725511	0.0000093	*GPR35 tv2, tv3*	G protein-coupled receptor 35	
		2_241554018_C_T_b37	rs56086919	0.0000093	*GPR35 tv2, tv3*	G protein-coupled receptor 35	
		2_241556826_A_C_b37	rs17846976	0.0000093	*GPR35 tv2, tv3*	G protein-coupled receptor 35	
		2_241558134_A_G_b37	rs73005895	0.0000093	*GPR35 tv2, tv3*	G protein-coupled receptor 35	
		2_241523790_G_A_b37	rs144304188	0.000010	non-coding variant of *CAPN10*	Calpain-10	
		2_241579108_G_T_b37	rs4676406	0.000019	non-coding region of *GPR35*	G protein-coupled receptor 35	ulcerative colitis, autoimmune thyroid disease, type I diabetes mellitus, common variable immunodeficiency, chronic childhood arthritis, ankylosing spondylitis, psoriasis, celiac disease, Crohn's disease, autoimmune disease, systemic lupus erythematosus, sclerosing cholangitis
**Muscle - Skeletal**	ENSG00000142330.15	***CAPN10***	3,75E-06	3,28E-02	4,93E-02		-0.233512
		**Variant ID**	**SNP ID**	**P-Value**	**Gene (tv or non-coding region) bearing the SNP**	**Full name of the gene**	**Known disease/trait association (GWAS catalog/SNPedia)**
		2_241536692_G_A_b37	rs3792275	3.7e-11	*CAPN10 tv1, tv3*	Calpain-10	
		2_241540110_A_G_b37	rs12616820	4.2e-11	non-coding region of *CAPN10*	Calpain-10	
		2_241527282_G_A_b37	rs67940783	5.1e-11	*CAPN10 tv1, tv3*	Calpain-10	
		2_241539003_A_G_b37	rs2953168	7.0e-11	*CAPN10 tv1, tv3*	Calpain-10	
		2_241536126_A_G_b37	rs7607759	8.6e-11	*CAPN10 tv1, tv3*	Calpain-10	
		2_241526053_A_T_b37	rs56352308	9.7e-11	*CAPN10 tv1, tv3*	Calpain-10	
		2_241526167_G_A_b37	rs56244291	9.7e-11	*CAPN10 tv1, tv3*	Calpain-10	
		2_241527462_G_T_b37	rs7596933	9.7e-11	*CAPN10 tv1, tv3*	Calpain-10	
		2_241528011_G_A_b37	rs12619657	9.7e-11	*CAPN10 tv1, tv3*	Calpain-10	
		2_241528927_G_A_b37	rs55893551	9.7e-11	*CAPN10 tv1, tv3*	Calpain-10	
		2_241528968_G_C_b37	rs55976923	9.7e-11	*CAPN10 tv1, tv3*	Calpain-10	
		2_241530616_A_T_b37	rs2975757	9.7e-11	*CAPN10 tv1, tv3*	Calpain-10	
		2_241531163_T_C_b37	rs2975760	9.7e-11	*CAPN10 tv1, tv3*	Calpain-10	
		2_241531479_A_G_b37	rs3792269	9.7e-11	*CAPN10 tv1, tv3*	Calpain-10	
		2_241532559_G_C_b37	rs41266975	9.7e-11	*CAPN10 tv1, tv3*	Calpain-10	
		2_241532620_C_A_b37	rs56783920	9.7e-11	*CAPN10 tv1, tv3*	Calpain-10	
		2_241533030_T_C_b37	rs3792273	9.7e-11	*CAPN10 tv1, tv3*	Calpain-10	
		2_241534163_G_A_b37	rs17846975	9.7e-11	*CAPN10 tv1, tv3*	Calpain-10	
		2_241536819_C_A_b37	rs3828345	9.7e-11	*CAPN10 tv1, tv3*	Calpain-10	

2_241537047_C_T_b37	rs56960757	9.7e-11	*CAPN10 tv1, tv3*	Calpain-10	
2_241537569_T_C_b37	rs17846981	9.7e-11	*CAPN10 tv1, tv3*	Calpain-10	
2_241538924_C_G_b37	rs67211173	9.7e-11	*CAPN10 tv1, tv3*	Calpain-10	
2_241526862_C_T_b37	rs67448739	1.0e-10	*CAPN10 tv1, tv3*	Calpain-10	
2_241526259_T_A_b37	rs55650580	1.1e-10	*CAPN10 tv1, tv3*	Calpain-10	
2_241536053_C_T_b37	rs2953171	1.3e-10	*CAPN10 tv1, tv3*	Calpain-10	
2_241538061_T_C_b37	rs55878652	1.4e-10	*CAPN10 tv1, tv3*	Calpain-10	
2_241528389_G_A_b37	rs55999124	1.7e-10	*CAPN10 tv1, tv3*	Calpain-10	
2_241539478_C_T_b37	rs57337179	2.5e-10	non-coding region of *CAPN10*	Calpain-10	
2_241539683_T_C_b37	rs67745756	3.2e-10	non-coding region of *CAPN10*	Calpain-10	
2_241526954_G_T_b37	rs112052223	6.4e-8	*CAPN10 tv1, tv3*	Calpain-10	
2_241541536_C_T_b37	rs2953163	1.7e-7	non-coding region		
2_241542801_C_G_b37	rs75659535	0.0000011	non-coding region		
2_241539264_C_T_b37	rs113940744	0.0000016	non-coding region of *CAPN10*	Calpain-10	
2_241534188_A_T_b37	rs70961719	0.0000030	*CAPN10 tv1, tv3*	Calpain-10	
2_241544852_A_C_b37	rs7565704	0.0000030	*GPR35 tv2, tv3*	G protein-coupled receptor 35	
2_241574401_G_A_b37	rs4676408	0.0000031	non-coding region of *GPR35*	G protein-coupled receptor 35	Inflammatory bowel disease, autoimmune thyroid disease, type I diabetes mellitus, Common variable immunodeficiency, chronic childhood arthritis, ankylosing spondylitis, psoriasis, celiac disease, ulcerative colitis, Crohn's disease, autoimmune disease, systemic lupus erythematosus
2_241478118_G_A_b37	rs113293453	0.0000032	*ANKMY1 tv1, tv2, tv3, tv4, tv5, tv6*	Ankyrin Repeat and MYND Domain Containing 1	
2_241543110_C_T_b37	rs75825616	0.0000037	*GPR35 tv2, tv3*	G protein-coupled receptor 35	
2_241516241_C_T_b37	rs76791955	0.0000053	*RNPEPL1*	Arginyl aminopeptidase like 1	
2_241517847_CAG_C_b37	rs3833579	0.0000053	*RNPEPL1*	Arginyl aminopeptidase like 1	
2_241518105_G_C_b37	rs1127207	0.0000053	*RNPEPL1*	Arginyl aminopeptidase like 1	
2_241518998_A_G_b37	rs72999942	0.0000053	non-coding region		
2_241523664_G_A_b37	rs139537724	0.0000053	non-coding variant of *CAPN10*	Calpain-10	
2_241524208_T_C_b37	rs7604368	0.0000053	non-coding variant of *CAPN10*	Calpain-10	

2_241525022_G_A_b37	rs72999959	0.0000053	non-coding variant of *CAPN10*	Calpain-10	
2_241525599_GA_G_b37	rs3833580	0.0000053	non-coding variant of *CAPN10*	Calpain-10	
2_241525840_C_T_b37	rs56373697	0.0000053	*CAPN10 tv1, tv3*	Calpain-10	
2_241529078_G_C_b37	rs56351903	0.0000053	*CAPN10 tv1, tv3*	Calpain-10	
2_241531575_A_G_b37	rs3792270	0.0000053	*CAPN10 tv1, tv3*	Calpain-10	
2_241531954_G_A_b37	rs56027088	0.0000053	*CAPN10 tv1, tv3*	Calpain-10	
2_241533934_T_C_b37	rs17846973	0.0000053	*CAPN10 tv1, tv3*	Calpain-10	
2_241535036_C_T_b37	rs73001804	0.0000053	*CAPN10 tv1, tv3*	Calpain-10	
2_241539071_T_C_b37	rs73001880	0.0000053	non-coding variant of *CAPN10*	Calpain-10	
2_241531117_A_C_b37	rs41266971	0.0000057	*CAPN10 tv1, tv3*	Calpain-10	
2_241540849_G_A_b37	rs113443535	0.0000057	non-coding region		
2_241579108_G_T_b37	rs4676406	0.0000057	non-coding region of *GPR35*	G protein-coupled receptor 35	ulcerative colitis, autoimmune thyroid disease, type I diabetes mellitus, common variable immunodeficiency, chronic childhood arthritis, ankylosing spondylitis, psoriasis, celiac disease, Crohn's disease, autoimmune disease, systemic lupus erythematosus, sclerosing cholangitis
2_241527785_C_A_b37	rs111280407	0.0000071	*CAPN10 tv1, tv3*	Calpain-10	
2_241528221_G_T_b37	rs77622920	0.0000071	*CAPN10 tv1, tv3*	Calpain-10	
2_241542183_C_T_b37	rs113909935	0.0000088	non-coding region		
2_241542703_C_T_b37	rs5030952	0.0000094	non-coding variant of *CAPN10*	Calpain-10	type II diabetes mellitus, polycystic ovary syndrome
2_241516094_G_C_b37	rs34344571	0.000011	*RNPEPL1*	Arginyl aminopeptidase like 1	
2_241552154_C_T_b37	rs73005864	0.000011	*GPR35 tv2, tv3*	G protein-coupled receptor 35	
2_241523790_G_A_b37	rs144304188	0.000012	non-coding variant of *CAPN10*	Calpain-10	
2_241558134_A_G_b37	rs73005895	0.000013	*GPR35 tv2, tv3*	G protein-coupled receptor 35	
2_241540229_G_T_b37	rs60001881	0.000013	non-coding region of *CAPN10*	Calpain-10	
2_241539943_C_T_b37	rs74733171	0.000013	non-coding region of *CAPN10*	Calpain-10	
2_241541129_A_T_b37	rs73003705	0.000013	non-coding region		
2_241541150_G_C_b37	rs73003707	0.000013	non-coding region		
2_241541811_C_T_b37	rs114966520	0.000013	non-coding region		
2_241543594_G_A_b37	rs73003723	0.000013	*GPR35 tv2, tv3*	G protein-coupled receptor 35	
2_241554018_C_T_b37	rs56086919	0.000013	*GPR35 tv2, tv3*	G protein-coupled receptor 35	
2_241552482_C_T_b37	rs73005866	0.000016	*GPR35 tv2, tv3*	G protein-coupled receptor 35	
2_241546271_T_G_b37	rs6725511	0.000016	*GPR35 tv2, tv3*	G protein-coupled receptor 35	

		2_241556826_A_C_b37	rs17846976	0.000017	*GPR35 tv2, tv3*	G protein-coupled receptor 35	
		2_241514665_G_A_b37	rs532703999	0.000019	*RNPEPL1*	Arginyl aminopeptidase like 1	
		2_241543863_C_T_b37	rs66815376	0.000022	*GPR35 tv2, tv3*	G protein-coupled receptor 35	
		2_241525639_A_C_b37	rs3749163	0.000030	*CAPN10 tv1, tv3*	Calpain-10	
		2_241526556_G_T_b37	rs60704894	0.000030	non-coding region of *CAPN10*	Calpain-10	
		2_241530793_A_G_b37	rs2975759	0.000030	*CAPN10 tv1, tv3*	Calpain-10	
		2_241553449_A_G_b37	rs56026499	0.000033	*GPR35 tv2, tv3*	G protein-coupled receptor 35	
		2_241510517_C_G_b37	rs73108015	0.000038	*RNPEPL1*	Arginyl aminopeptidase like 1	
**Whole Blood**	ENSG00000142330.15	***CAPN10***	5,04E-03	0.000238092	0.000363852		-0.152938
		**Variant ID**	**SNP ID**	**P-Value**	**Gene (tv or non-coding region) bearing the SNP**	**Full name of the gene**	**Known disease/trait association (GWAS catalog/SNPedia)**
		2_241538061_T_C_b37	rs55878652	5.0e-8	*CAPN10 tv1, tv3*	Calpain-10	
		2_241536692_G_A_b37	rs3792275	7.2e-8	*CAPN10 tv1, tv3*	Calpain-10	
		2_241536126_A_G_b37	rs7607759	8.7e-8	*CAPN10 tv1, tv3*	Calpain-10	
		2_241539003_A_G_b37	rs2953168	9.3e-8	*CAPN10 tv1, tv3*	Calpain-10	
		2_241527282_G_A_b37	rs67940783	9.8e-8	*CAPN10 tv1, tv3*	Calpain-10	
		2_241531163_T_C_b37	rs2975760	1.0e-7	*CAPN10 tv1, tv3*	Calpain-10	
		2_241533030_T_C_b37	rs3792273	1.0e-7	*CAPN10 tv1, tv3*	Calpain-10	
		2_241534163_G_A_b37	rs17846975	1.0e-7	*CAPN10 tv1, tv3*	Calpain-10	
		2_241536819_C_A_b37	rs3828345	1.0e-7	*CAPN10 tv1, tv3*	Calpain-10	
		2_241537569_T_C_b37	rs17846981	1.0e-7	*CAPN10 tv1, tv3*	Calpain-10	
		2_241538924_C_G_b37	rs67211173	1.0e-7	*CAPN10 tv1, tv3*	Calpain-10	
		2_241531479_A_G_b37	rs3792269	1.0e-7	*CAPN10 tv1, tv3*	Calpain-10	
		2_241532559_G_C_b37	rs41266975	1.0e-7	*CAPN10 tv1, tv3*	Calpain-10	
		2_241532620_C_A_b37	rs56783920	1.0e-7	*CAPN10 tv1, tv3*	Calpain-10	
		2_241526053_A_T_b37	rs56352308	1.0e-7	*CAPN10 tv1, tv3*	Calpain-10	
		2_241526167_G_A_b37	rs56244291	1.0e-7	*CAPN10 tv1, tv3*	Calpain-10	
		2_241527462_G_T_b37	rs7596933	1.0e-7	*CAPN10 tv1, tv3*	Calpain-10	
		2_241528011_G_A_b37	rs12619657	1.0e-7	*CAPN10 tv1, tv3*	Calpain-10	
		2_241528927_G_A_b37	rs55893551	1.0e-7	*CAPN10 tv1, tv3*	Calpain-10	
		2_241528968_G_C_b37	rs55976923	1.0e-7	*CAPN10 tv1, tv3*	Calpain-10	
		2_241530616_A_T_b37	rs2975757	1.0e-7	*CAPN10 tv1, tv3*	Calpain-10	
		2_241526259_T_A_b37	rs55650580	1.0e-7	*CAPN10 tv1, tv3*	Calpain-10	
		2_241528389_G_A_b37	rs55999124	1.5e-7	*CAPN10 tv1, tv3*	Calpain-10	
		2_241536053_C_T_b37	rs2953171	1.6e-7	*CAPN10 tv1, tv3*	Calpain-10	
		2_241537047_C_T_b37	rs56960757	1.8e-7	*CAPN10 tv1, tv3*	Calpain-10	

	2_241526862_C_T_b37	rs67448739	1.8e-7	*CAPN10 tv1, tv3*	Calpain-10
	2_241539478_C_T_b37	rs57337179	2.0e-7	non-coding region of *CAPN10*	Calpain-10
	2_241539683_T_C_b37	rs67745756	2.0e-7	non-coding region of *CAPN10*	Calpain-10
	2_241540110_A_G_b37	rs12616820	2.3e-7	non-coding region of *CAPN10*	Calpain-10
	2_241540229_G_T_b37	rs60001881	0.0000047	non-coding region of *CAPN10*	Calpain-10
	2_241543863_C_T_b37	rs66815376	0.000018	*GPR35 tv2, tv3*	G protein-coupled receptor 35

Supplemental Figure 1. Diagram describing the methodological processes of RNA-Seq and RT-qPCR assays applied. Total RNA was isolated from 0.4 ml peripheral blood samples using the NucleoSpin RNA Blood kit (MACHEREY-NAGEL GmbH & Co. KG, Düren, Germany) upon direct blood lysis, according to manufacturer's instructions. ***For RNA-Seq***: RNA-Seq was performed in peripheral blood specimens from representative patients (n=4) and CTs (n=2). 500 ng of high-quality total RNA was used for the preparation of cDNA libraries. Fragmentation and purification of the poly(A) RNA, and construction and purification of the libraries were performed using the Ion Total RNA-Seq Kit v2 for Whole Transcriptome Libraries (Ion Torrent by Life technologies, Thermo Fisher Scientific), as per manufacturer's instructions. The size distribution of the isolated RNA and amplified cDNA samples were assessed in an Agilent 2100 Bioanalyzer using the RNA 6000 Pico Kit and the Agilent High Sensitivity DNA Kit, respectively (all from Agilent Technologies, Santa Clara, CA, USA). Templates for next-generation sequencing (NGS) were prepared using the Ion PGM Template OT2 200 Kit in an Ion OneTouch 2 System (Ion Torrent), following the instructions of the manufacturer. The quality of the template-positive Ion Sphere Particles was assessed with the Ion Sphere Quality Control Kit in a Qubit 2.0 Fluorometer (Invitrogen). The enrichment of the template-positive Ion Sphere Particles was performed in an Ion OneTouch ES instrument using the Ion PGM Template OT2 200 Kit (Ion Torrent), according to the manufacturer's instructions. NGS was carried out in an Ion Personal Genome Machine (PGM) System, using the Ion PGM Sequencing 200 Kit v2 and Ion 316 Chips (one chip per sample) (Ion Torrent), as per manufacturer's instructions. Analysis of RNA-seq raw data was performed using the Torrent Suite Software (Ion Torrent). Normalized values (reads per kilobase million, RPKMs) were produced for each sample. Mean of raw data (reads), of RPKMs, % coefficient of variation (CV) and standard deviation (SD) values in CT and T2D groups were estimated for all transcript variants identified (data not shown). ***For RT-qPCR assays***: 0.5 μg of total RNA was reverse-transcribed to cDNA in a 20-μl reaction containing 6.67 μM oligo-dT primer, 200 U MMLV reverse transcriptase, 40 U recombinant ribonuclease inhibitor and 0.5 mM dNTPs mix (all from Invitrogen by Thermo Fischer Scientific, Waltham, MA, USA), at 37 °C for 50 min, following 15 min at 70 °C. SYBR-Green fluorescent-based qPCR assays were specifically developed for the amplification of the mRNA of the genes-of-interest, or certain transcript variants of them found to be differentially expressed between T2D patients and CTs in RNA-seq experiments. qPCR reactions were performed in duplicates in a 7500 Real-Time PCR System (Applied Biosystems, Carlsbad, CA, USA). The 10-μl reaction mixture contained 5 ng of cDNA template, 5 μL of Kapa SYBR Fast Universal 2X qPCR Master Mix (Kapa Biosystems, Inc., Woburn, MA, USA) and optimal quantity of each primer (Supplemental Table 1). The thermal protocol was: 95 °C for 3 min, 95 °C for 3 sec (40 cycles), 60 °C for 30 sec. The $2^{-\Delta\Delta CT}$ relative quantification (RQ) method was applied for the estimation of mRNA levels. The human hypoxanthine phosphoribosyltransferase 1 (*HPRT1*) gene was selected to be used as endogenous reference gene, upon pilot comparative experiments (data not shown), and the 1.2B4 human immortalized beta-pancreatic cell line (ECACC, Salisbury, UK) as calibrator.

Supplemental Figure 2. Representative curves for the amplification of total transcript variants of the *HPRT1* gene (NM_000194.2; the only variant), *CDK5* gene (NM_004935.3, NM_001164410.2), and transcript variant (tv) 2 of the *CDK5* gene (NM_001164410.2) (***upper row of panels***). Corresponding melting curves used for the verification of the specificity of the amplicons (***middle row***), and standard curves indicating the % efficiency of the developed qPCR assays (***lower row***). The % efficiency was estimated using the slope of the curve and the qPCR Efficiency Calculator online software (Thermo Fisher Scientific, Waltham, MA, U.S.A.). In each case, the efficiency of the developed protocol was ≥90,46% and the amplification factor 1.90-2.10.

Supplemental Figure 1

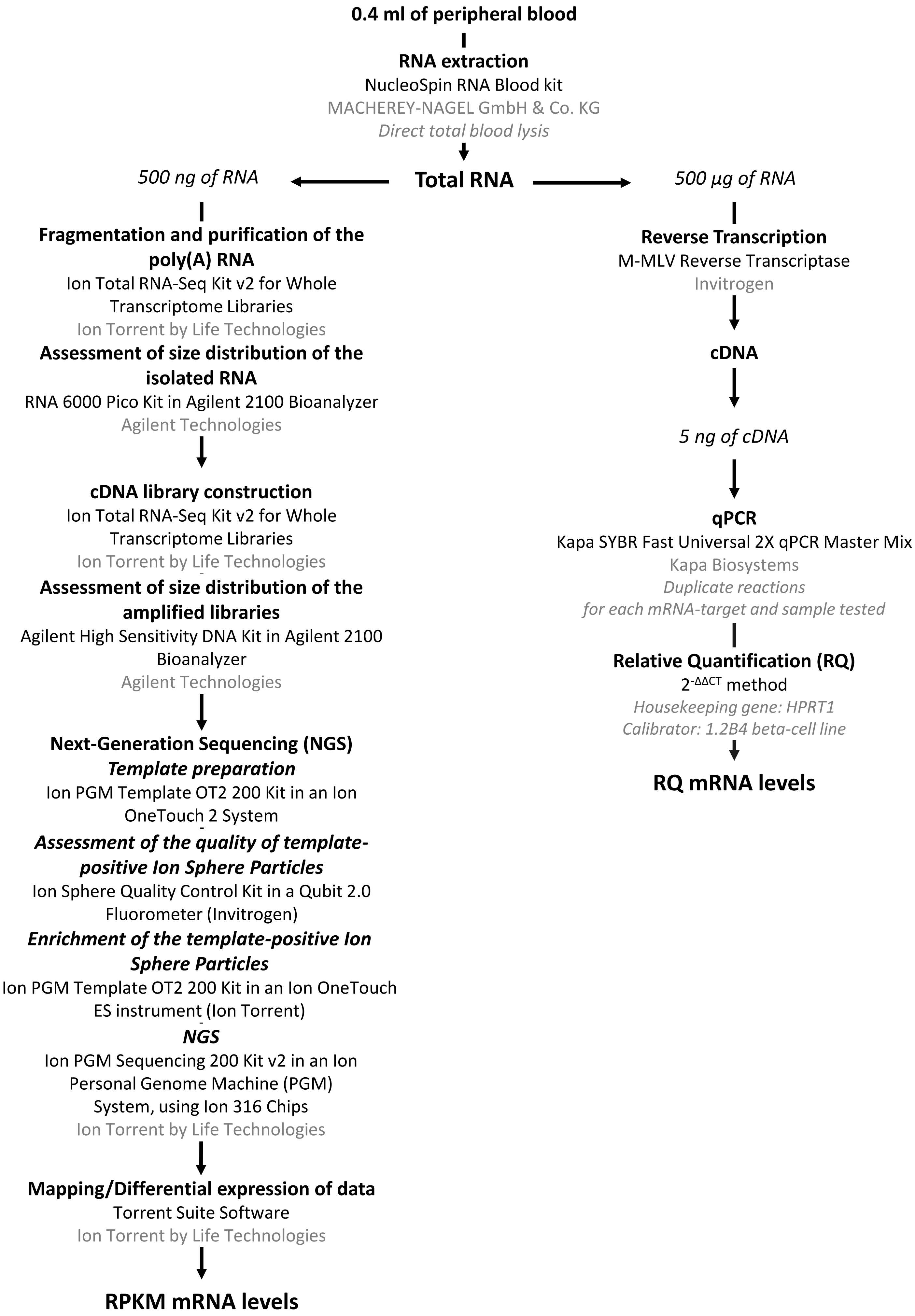
0.4 ml of peripheral blood
RNA extraction
NucleoSpin RNA Blood kit
MACHEREY-NAGEL GmbH & Co. KG
Direct total blood lysis
Total RNA
500 ng of RNA
Fragmentation and purification of the poly(A) RNA
Ion Total RNA-Seq Kit v2 for Whole Transcriptome Libraries
Ion Torrent by Life Technologies
Assessment of size distribution of the isolated RNA
RNA 6000 Pico Kit in Agilent 2100 Bioanalyzer
Agilent Technologies
cDNA library construction
Ion Total RNA-Seq Kit v2 for Whole Transcriptome Libraries
Ion Torrent by Life Technologies
Assessment of size distribution of the amplified libraries
Agilent High Sensitivity DNA Kit in Agilent 2100 Bioanalyzer
Agilent Technologies
Next-Generation Sequencing (NGS)
Template preparation
Ion PGM Template OT2 200 Kit in an Ion OneTouch 2 System
Assessment of the quality of template-positive Ion Sphere Particles
Ion Sphere Quality Control Kit in a Qubit 2.0 Fluorometer (Invitrogen)
Enrichment of the template-positive Ion Sphere Particles
Ion PGM Template OT2 200 Kit in an Ion OneTouch ES instrument (Ion Torrent)
NGS
Ion PGM Sequencing 200 Kit v2 in an Ion Personal Genome Machine (PGM) System, using Ion 316 Chips
Ion Torrent by Life Technologies
Mapping/Differential expression of data
Torrent Suite Software
Ion Torrent by Life Technologies
RPKM mRNA levels
500 µg of RNA
Reverse Transcription
M-MLV Reverse Transcriptase
Invitrogen
cDNA
5 ng of cDNA
qPCR
Kapa SYBR Fast Universal 2X qPCR Master Mix
Kapa Biosystems
Duplicate reactions
for each mRNA-target and sample tested
Relative Quantification (RQ)
$2^{-\Delta\Delta CT}$ method
Housekeeping gene: HPRT1
Calibrator: 1.2B4 beta-cell line
RQ mRNA levels

Supplemental Figure 2

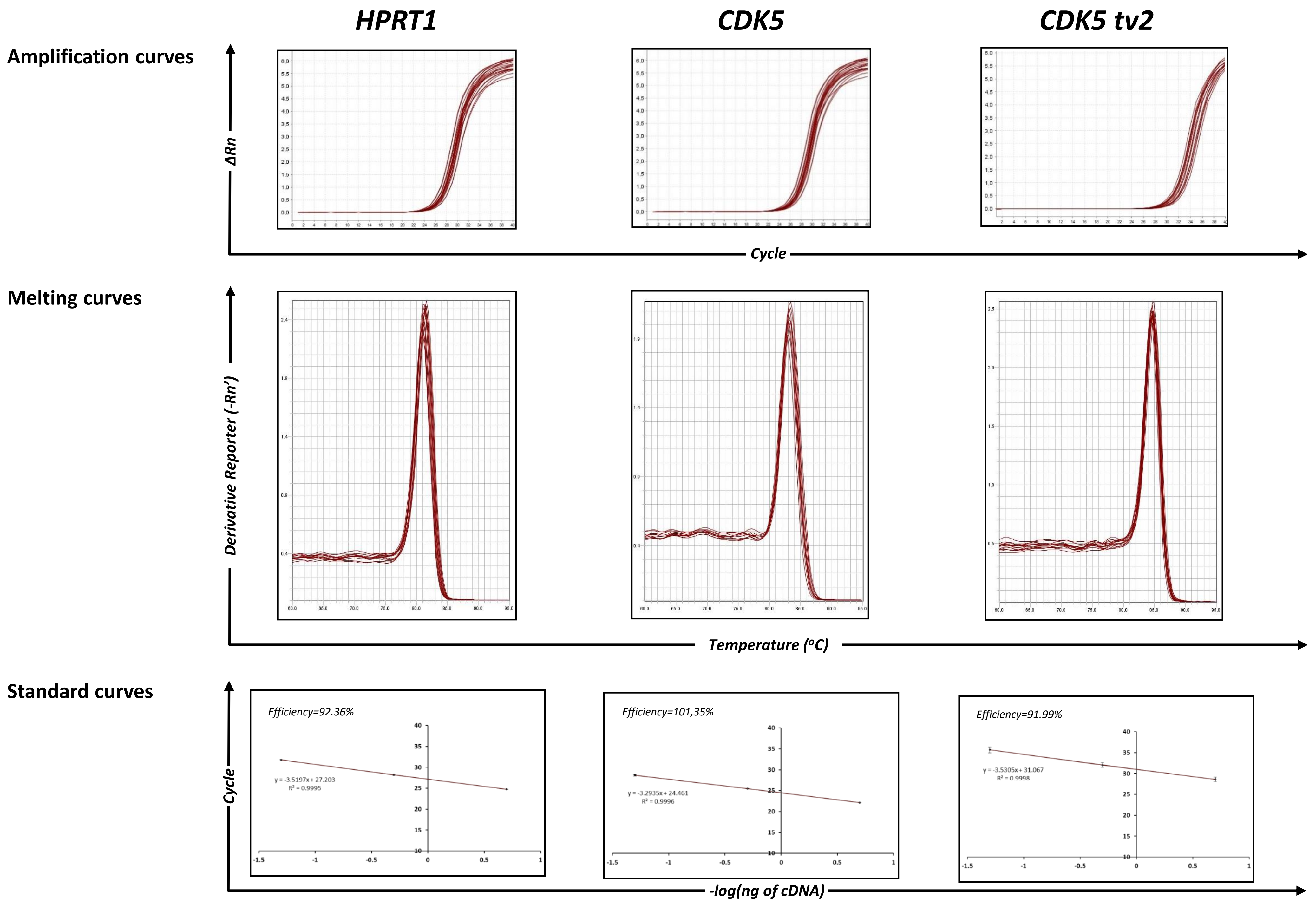
HPRT1
CDK5
CDK5 tv2
Amplification curves
ΔRn
Cycle
Melting curves
Derivative Reporter (-Rn')
Temperature (°C)
Standard curves
Cycle
-log(ng of cDNA)
Efficiency=92.36%
y = -3.5197x + 27.203
R² = 0.9995
Efficiency=101,35%
y = -3.2935x + 24.461
R² = 0.9996
Efficiency=91.99%
y = -3.5305x + 31.067
R² = 0.9998
